# A randomised, double-blind, placebo-controlled, pilot trial of intravenous plasma purified alpha-1 antitrypsin for SARS-CoV-2-induced Acute Respiratory Distress Syndrome: a structured summary of a study protocol for a randomised, controlled trial

**DOI:** 10.1186/s13063-021-05254-0

**Published:** 2021-04-19

**Authors:** Natalie L. McEvoy, Jennifer L. Clarke, Oliver J. Mc Elvaney, Oisin F. Mc Elvaney, Fiona Boland, Deirdre Hyland, Pierce Geoghegan, Karen Donnelly, Oisin Friel, Ailbhe Cullen, Ann M. Collins, Daniel Fraughen, Ignacio Martin-Loeches, Martina Hennessy, John G. Laffey, Noel G. Mc Elvaney, Gerard F. Curley

**Affiliations:** 1 Royal College of Surgeons in Ireland, Dublin, Ireland; 2 St James’ University Hospital, Dublin, Ireland; 3 Galway University Hospital, Galway, Ireland

**Keywords:** SARS-CoV-2, Covid-19, randomised controlled trial, protocol, Acute Respiratory Distress Syndrome, ARDS, alpha-1 antitrypsin

## Abstract

**Objectives:**

The primary objective is to demonstrate that, in patients with PCR-confirmed SARS-CoV-2 resulting in Acute Respiratory Distress Syndrome (ARDS), administration of 120mg/kg of body weight of intravenous Prolastin®(plasma-purified alpha-1 antitrypsin) reduces circulating plasma levels of interleukin-6 (IL-6). Secondary objectives are to determine the effects of intravenous Prolastin® on important clinical outcomes including the incidence of adverse events (AEs) and serious adverse events (SAEs).

**Trial design:**

Phase 2, randomised, double-blind, placebo-controlled, pilot trial.

**Participants:**

The study will be conducted in Intensive Care Units in hospitals across Ireland. Patients with a laboratory-confirmed diagnosis of SARS-CoV-2-infection, moderate to severe ARDS (meeting Berlin criteria for a diagnosis of ARDS with a PaO_2_/FiO_2_ ratio <200 mmHg), >18 years of age and requiring invasive or non-invasive mechanical ventilation. All individuals meeting any of the following exclusion criteria at baseline or during screening will be excluded from study participation: more than 96 hours has elapsed from onset of ARDS; age < 18 years; known to be pregnant or breastfeeding; participation in a clinical trial of an investigational medicinal product (other than antibiotics or antivirals) within 30 days; major trauma in the prior 5 days; presence of any active malignancy (other than nonmelanoma skin cancer) which required treatment within the last year; WHO Class III or IV pulmonary hypertension; pulmonary embolism prior to hospital admission within past 3 months; currently receiving extracorporeal life support (ECLS); chronic kidney disease receiving dialysis; severe chronic liver disease with Child-Pugh score > 12; DNAR (Do Not Attempt Resuscitation) order in place; treatment withdrawal imminent within 24 hours; Prisoners; non-English speaking patients or those who do not adequately understand verbal or written information unless an interpreter is available; IgA deficiency.

**Intervention and comparator:**

Intervention: Either a once weekly intravenous infusion of Prolastin® at 120mg/kg of body weight for 4 weeks or a single dose of Prolastin® at 120mg/kg of body weight intravenously followed by once weekly intravenous infusion of an equal volume of 0.9% sodium chloride for a further 3 weeks. Comparator (placebo): An equal volume of 0.9% sodium chloride intravenously once per week for four weeks.

**Main outcomes:**

The primary effectiveness outcome measure is the change in plasma concentration of IL-6 at 7 days as measured by ELISA. Secondary outcomes include: safety and tolerability of Prolastin® in the respective groups (as defined by the number of SAEs and AEs); PaO_2_/FiO_2_ ratio; respiratory compliance; sequential organ failure assessment (SOFA) score; mortality; time on ventilator in days; plasma concentration of alpha-1 antitrypsin (AAT) as measured by nephelometry; plasma concentrations of interleukin-1β (IL-1β), interleukin-8 (IL-8), interleukin-10 (IL-10), soluble TNF receptor 1 (sTNFR1, a surrogate marker for TNF-α) as measured by ELISA; development of shock; acute kidney injury; need for renal replacement therapy; clinical relapse, as defined by the need for readmission to the ICU or a marked decline in PaO_2_/FiO_2_ or development of shock or mortality following a period of sustained clinical improvement; secondary bacterial pneumonia as defined by the combination of radiographic findings and sputum/airway secretion microscopy and culture.

**Randomisation:**

Following informed consent/assent patients will be randomised. The randomisation lists will be prepared by the study statistician and given to the unblinded trial personnel. However, the statistician will not be exposed to how the planned treatment will be allocated to the treatment codes. Randomisation will be conducted in a 1:1:1 ratio, stratified by site and age.

**Blinding (masking):**

The investigator, treating physician, other members of the site research team and patients will be blinded to treatment allocation. The clinical trial pharmacy personnel and research nurses will be unblinded to facilitate intervention and placebo preparation. The unblinded individuals will keep the treatment information confidential. The infusion bag will be masked at the time of preparation and will be administered via a masked infusion set to maintain blinding.

**Numbers to be randomised (sample size):**

A total of 36 patients will be recruited and randomised in a 1:1:1 ratio to each of the trial arms.

**Trial status:**

In March 2020, version 1.0 of the trial protocol was submitted to the local research ethics committee (REC), Health Research Consent Declaration Committee (HRCDC) and the Health Products regulatory Authority (HPRA). REC approval was granted on April 1^st^ 2020, HPRA approval was granted on April 24^th^ 2020 and the HRCDC provided a conditional declaration on April 17^th^ 2020. In July 2020 a substantial amendment (version 2.0) was submitted to the REC, HRCDC and HPRA. Protocol changes in this amendment included: the addition of trial sites; extending the duration of the trial to 12 months from 3 months; removal of inclusion criteria requiring the need for vasopressors; amendment of randomisation schedule to stratify by age only and not BMI and sex; correction of grammatical error in relation to infusion duration; to allow for inclusion of subjects who may have been enrolled in a clinical trial involving either antibiotics or anti-virals in the past 30 days; to allow for inclusion of subjects who may be currently enrolled in a clinical trial involving either antibiotics or anti-virals; to remove the need for exclusion based on alpha-1 antitrypsin phenotype; removal of mandatory isoelectric focusing of plasma to confirm Pi*MM status at screening; removal of need for mandatory echocardiogram at screening; amendment on procedures around plasma analysis to reflect that this will be conducted at the central site laboratory (as trial is multi-site and no longer single site); wording amended to reflect that interim analysis of cytokine levels taken at 7 days may be conducted. HRCDC approved version 2.0 on September 14th 2020, and HPRA approved on October 22nd 2020. REC approved the substantial amendment on November 23^rd^. In November 2020, version 3.0 of the trial protocol was submitted to the REC and HPRA. The rationale for this amendment was to allow for patients with moderate to severe ARDS from SARS-CoV-2 with non-invasive ventilation. HPRA approved this amendment on December 1st 2020 and the REC approved the amendment on December 8th 2020. Patient recruitment commenced in April 2020 and the last patient will be recruited to the trial in April 2021. The last visit of the last patient is anticipated to occur in April 2021. At time of writing, patient recruitment is now complete, however follow-up patient visits and data collection are ongoing.

**Trial registration:**

EudraCT 2020-001391-15 (Registered 31 Mar 2020).

**Full protocol:**

The full protocol (version 3.0 23.11.2020) is attached as an additional file accessible from the Trials website (Additional file 1). In the interest in expediting dissemination of this material, the familiar formatting has been eliminated; this Letter serves as a summary of the key elements of the full protocol.

The study protocol has been reported in accordance with the Standard Protocol Items: Recommendations for Clinical Interventional Trials (SPIRIT) guidelines (Additional file 2).

**Supplementary Information:**

The online version contains supplementary material available at 10.1186/s13063-021-05254-0.

## Supplementary Information

**Additional file 1.** Full study protocol.

**Additional file 2.** SPIRIT 2013 Checklist: Recommended items to address in a clinical trial protocol and related documents*.

## Data Availability

The datasets generated and/or analysed during the current study are not publicly available due to relevant data protection laws but may be available from the corresponding author on reasonable request.

