# Supplementary material for: A randomised, double-blind, placebo-controlled, pilot trial of intravenous plasma purified alpha-1 antitrypsin for SARS-CoV-2-induced Acute Respiratory Distress Syndrome: a structured summary of a study protocol for a randomised, controlled trial
Source: Trials. 2021 Apr 19;22:288. doi: 10.1186/s13063-021-05254-0 (PMC8054126; doi:10.1186/s13063-021-05254-0)
Supplement: Supplementary file 1 — Additional file 1. Full study protocol. [file 13063_2021_5254_MOESM1_ESM.pdf]

Version Number	Version Date	Summary of Revisions Made:
3.0	23-Nov-2020	

1. Study Title

**Full title of trial**	A randomized double-blind placebo-controlled, pilot trial of intravenous plasma-purified alpha-1 antitrypsin for severe COVID-19 illness.
**Short title**	A randomized double-blind placebo-controlled, pilot trial of intravenous plasma-purified alpha-1 antitrypsin for severe COVID-19 illness.
**Version and date of protocol:**	Version 3.0, 23-Nov-2020
**Sponsor:**	Royal College of Surgeons Ireland (RCSI)
**Funder (s) :**	
**EudraCT no**	2020-001391-15
**ACTIVE IMP(s):**	Prolastin
**PLACEBO IMP(s):**	0.9% sodium chloride
**Phase of trial**	Phase 2
**Sites(s)**	Multi-site *(see Section 8 for details*)

Chief investigator:
Prof. Ger Curley
RCSI Professor of Anaesthesia and Critical Care, Beaumont Hospital, Beaumont Hospital, Beaumont Rd, Dublin 9, Ireland.
Tel: : +353 1 8093810
Fax: +353 1 8093755
Email: gercurley@rcsi.ie

Sponsor Representative:
Dr Muiris Dowling RCSI Sponsor Officer 2nd Floor Ardilaun House, Block B, 111 St. Stephen's Green, Dublin 2, Ireland,
Tel: : +353 87 6245669Fax: +353 1 402-2453
Email: mauricedowling@rcsi.ie

2. Sponsor and Chief Investigator Signature Page

The Chief Investigator and sponsor representatives have discussed this protocol. The investigators agree to perform the investigations and to abide by this protocol
The investigator agrees to conduct the trial in compliance with the approved protocol, EU Directives 2001/20/EC and 2005/28/EC, Statutory instruments 190 2004 and 374 2006, Data Protection Act 1988 and (amendment) 2003 and ICH GCP

Chief investigator

Prof Ger Curley

Gerard Curley (Dec 8, 2020 21:01 GMT) Dec 8, 2020

Signature Date

Sponsor Representative

Dr Muiris Dowling

Maurice Dowling
Maurice Dowling (Dec 9, 2020 07:58 GMT) Dec 9, 2020

Signature Date

3. Principal Investigator Signature Page

To be signed and returned to Sponsor,

- The signature below constitutes the approval of this protocol and attests its content, abiding by the above-mentioned version and any subsequent amendments during the participation in the trial.
- The signature represents the necessary assurances that this trial will be conducted according to all stipulations outlined in the protocol, including all statements regarding confidentiality, and in accordance with ICH GCP, regulatory requirements and the Declaration on Helsinki.
- This signature confirms that I have no direct or indirect involvement in the outcome of the clinical trial that could in anyway be regarded as a possible conflict of interest

Principal investigator

(name and contact details)

____

Signature Date

Table of Contents

1. Study Title 1
2. Sponsor and Chief Investigator Signature Page 2
3. Principal Investigator Signature Page 3
4. List of abbreviations 7
5. Trial personnel 8
6. Sponsor Personnel 9
7. Summary 10
8. Key Roles and Contact Information 16
9. Introduction 18

9.1 Background 18
9.2 Preclinical data 20
9.3 Clinical data 21
9.4 Rationale and risks/benefits 22
9.5 Assessment and management of risk 23
9.6 Potential Risks 25
9.7 Potential Benefits 27
10 Objectives 27
10.1 Primary Objective(s) 27
10.2 Secondary Objective(s) 27
10.3 Study Outcome Measures 28
10.3.1 Primary outcome measure 28
10.3.2 Secondary outcome measure 28
11 Trial design 29
11.1 Overall design 29
11.2 Sub-studies (if applicable) 31
12 Selection of Subjects 31
12.1 Inclusion criteria 31
12.2 Exclusion criteria 32
13 Recruitment 32
14 Study procedures and schedule of assessments 32
14.1 Informed consent procedure 32
14.2 Randomisation procedures 33
14.3 Unblinding 33
14.3.1 Emergency Unblinding 33
14.3.2 Unblinding for the submission of SUSAR reports: 34
14.4 Screening Period 36
14.5 Baseline assessments 37
14.6 Treatment procedures 37
14.7 Subsequent assessments 38
14.8 Flowchart of study assessments 39
14.9 Methods 40
14.9.1 Laboratory procedures 40
14.10 Definition of end of trial 40
14.11 Discontinuation/withdrawal of participants and ‘stopping rules’ 40
15 Name and description of all drugs used in the trial 40

15.1 Treatment of subjects..40
15.2 Concomitant medication..41
16 Investigational Medicinal Product..41
16.1 Name and description of investigational medicinal product(s)......................41
16.2 Name and description of each NIMP 42
Not applicable...42
16.3 Summary of findings from non-clinical studies 42
16.4 Summary of findings from clinical studies..43
16.5 Summary of known and potential risks and benefits.....................................43
16.6 Description and justification of route of administration and dosage.............44
16.7 Dosages, dosage modifications and method of administration 44
16.8 Preparation and labelling of Investigational Medicinal Product 45
16.9 Drug accountability 46
16.10 Source of IMPs including placebo..46
16.11 Dose modifications 46
16.12 Assessment of compliance..46
16.13 Post-trial IMP arrangements..46
17 Recording and reporting of adverse events and reactions 46
17.1 Definitions 46
17.2 Recording adverse events 47
17.3 Assessments of Adverse Events 49
17.3.1 Severity.. 49
17.3.2 Causality.. 49
17.3.3 Expectedness 49
17.3.4 Seriousness.. 50
17.4 Procedures for recording and reporting Serious Adverse Events..................50
17.4.1 Non reportable events............................... **Error! Bookmark not defined.**
17.4.2 Notification of deaths 51
17.4.3 Reporting SUSARs.. 51
17.4.4 Development Safety Update Reports.. 51
17.4.5 Annual progress reports.. 51
17.4.6 Pregnancy 51
17.4.7 Overdose.. 51
17.4.8 Reporting Urgent Safety Measures 52
17.5 The type and duration of the follow-up of subjects after adverse events. 52
17.5.1 Notification of Serious Breaches to GCP and/or the protocol 52
18 Data management and quality assurance..52
18.1 Confidentiality..52
18.2 Data collection tools and source document identification.............................52
18.3 Data handling and analysis..53
19 Record keeping and archiving..53
20 Statistical Considerations 53
20.1 Outcomes..53
20.1.1 Primary outcomes.. 53
20.1.2 Secondary outcomes 54
20.2 Sample size and recruitment..54
20.2.1 Sample size calculation 54
21.2.2 Planned recruitment rate.. 54

20.3 Statistical analysis plan 55
20.3.1 Summary of baseline data and flow of patients 55
20.3.2 Primary outcome analysis 55
20.3.3 Secondary outcome analysis 55
20.3.4 Sensitivity and other planned analyses 55
20.4 Randomisation methods 55
20.5 Interim analysis 55
20.6 Other statistical considerations 56
21 Name of Committees involved in trial 56
22 Direct Access to Source Data/Documents 56
23 Ethics and regulatory requirements 56
24 Monitoring requirement for the trial 57
25 Finance 57
26 Insurance 57
27 Publication policy 57
28 Statement of compliance 58
29 Appendices 58
30 References 59

4. List of abbreviations

AE	Adverse Event
AR	Adverse Reaction
CA	Competent Authority
CI	Chief Investigator
CRF	Case Report Form
CTA	Clinical Trial Authorisation
CTIMP	Clinical Trial of Investigational Medicinal Product
DSMB	Data Safety Monitoring Board
DSUR	Development Safety Update Report
EMEA	European Medicines Agency
EU	European Union
EudraCT	European Clinical Trials Database
EudraVIGILANCE	European database for Pharmacovigilance
GCP	Good Clinical Practice
GMP	Good Manufacturing Practice
IB	Investigator Brochure
ICF	Informed Consent Form
IMP	Investigational Medicinal Product
IMPD	Investigational Medicinal Product Dossier
ISF	Investigator Site File
MA	Marketing Authorisation
PI	Principal Investigator
PIL	Participant Information Leaflet
QA	Quality Assurance
QC	Quality Control
QP	Qualified Person for release of trial drug
RCT	Randomised Control Trial
REC	Research Ethics Committee
SAR	Serious Adverse Reaction
SAE	Serious Adverse Event
SDV	Source Document Verification
SOP	Standard Operating Procedure
SmPC	Summary of Product Characteristics
SSA	Site Specific Assessment
SUSAR	Suspected Unexpected Serious Adverse Reaction
TMG	Trial Management Group
TSC	Trial Steering Committee

5. Trial personnel

Chief Investigator (CI) Prof. Ger Curley,
Professor of Anaesthesia and Critical Care,
Beaumont Hospital, Beaumont Rd, Dublin 9, Ireland.
e-mail: gercurley@rcsi.ie
tel: : +353 1 8093810 fax: +353 1 8093755

Co-Investigator(s) Noel G. McElvaney
Professor of Medicine
Department of Medicine
Royal College of Surgeons in Ireland (RCSI)
Education and Research Centre, Beaumont Hospital, Dublin 9
tel: +353 1 809 3763 E-mail: gmcelvaney@rcsi.ie

Statistician Fiona Boland
e-mail: fionaboland@rcsi.ie
tel: +3531-402-2507

6. Sponsor Personnel

Sponsor Representative	Maurice Dowling<br><br>e-mail: mauricedowling@rcsi.ie<br>tel: 0876245669
Quality & Regulatory Affairs Manager (QRAM)	Mandy Jackson,<br>Quality and Regulatory Affairs Manager,<br>RCSI Clinical Research Centre, RCSI ERC Building,<br>Beaumont Hospital, Dublin 9, Ireland.<br>e-mail: mandyjackson@rcsi.ie<br>tel +353 1 8093863 F: +353 1 809 3809
Pharmacovigilance Officer & Monitor	Angela Farrelly<br>Clinical Trials Manager/GCP Monitor<br>RCSI Clinical Research Centre, RCSI ERC Building<br>angelafarrelly@rcsi.com<br>tel +353 1 8093725 F: +353 1 809 3809

7. Summary

**Title:**	A randomized double-blind placebo-controlled, pilot trial of intravenous plasma-purified alpha-1 antitrypsin for severe COVID-19 illness.
**Trial medication:**	Prolastin
**Placebo**	0.9% sodium chloride
**Phase of trial:**	Phase 2
**Objectives:**	The primary objective is to demonstrate a biological effect of IV Prolastin administered weekly at 120mg per kilogram of body weight in patients with severe COVID-19 illness requiring intubation and mechanical ventilation for ARDS by reducing circulating levels of IL-6 as measured by plasma ELISA.

The secondary objectives of this study are:

1. To determine the safety and tolerability of [IV Prolastin administered once at 120mg per kilogram of body weight] and [IV Prolastin administered weekly at 120mg per kilogram of body weight for 4 weeks], as assessed by the number of AEs and SAEs.

   and to determine the effects of [IV Prolastin administered once at 120mg per kilogram of body weight] and [IV Prolastin administered weekly at 120mg per kilogram of body weight for 4 weeks] on:

2. Physiological indices of respiratory dysfunction reflecting severity of ARDS, as measured by oxygenation index (OI), respiratory compliance
3. Sequential organ failure assessment (SOFA) score, an assessment of clinical severity in critically unwell patients

4. Mortality, given that no treatment to date has been shown to influence this outcome
5. Time on ventilator in days, given the impending shortage of ICU bed availability
6. Circulating alpha-1 antitrypsin (AAT) levels
7. Circulating levels of IL-1β, IL-8, IL-10, soluble TNF receptor 1 (a surrogate marker for TNF-α), all of which have been implicated in pulmonary and systemic inflammation, and also to be suppressed by AAT in vivo
8. Development of shock, a key cause of death in this cohort
9. Acute kidney injury, a key cause of death in this cohort
10. Need for renal replacement therapy, given the impending shortage of renal replacement facilities for the population studied
11. Clinical relapse, since this must be clarified to facilitate safer discharge from an ICU setting
12. Length of ICU stay in days

Type of trial: Phase 2, randomized double-blind placebo-controlled, pilot trial

Trial design and methods: We propose a randomized, placebo-controlled, double blind, pilot parallel group study of [IV Prolastin administered once at 120mg per kilogram of body weight] versus [IV Prolastin administered weekly at 120mg per kilogram of body weight for 4 weeks] versus placebo. Both the active drug and matching placebo will be prepared outside of the ICU by unblinded trial personnel. The placebo is 0.9% sodium chloride solution for infusion.

Trial duration per participant: 5 weeks

**Estimated total trial duration:**	12 months
**Planned trial sites:**	Multi-site; Beaumont Hospital, Dublin 9, Ireland, Galway University Hospital, St James's University Hospital, Mater Misericordiae University Hospital.
**Total number of participants planned:**	36 participants
**Main inclusion/exclusion criteria:**	In order to be eligible to participate in this study, an individual must meet all of the following inclusion criteria:<br>1. Laboratory-confirmed diagnosis of COVID-19 infection<br>2. Moderate to severe ARDS with a PaO2/FiO2 ratio <200<br>3. >18 years of age<br>4. Patients receiving invasive mechanical ventilation or non-invasive ventilation<br><br>All individuals meeting any of these exclusion criteria at baseline or during screening will be excluded from study participation:<br>1. More than 96 hours from the onset of ARDS<br>2. Age < 18 years<br>3. Known to be pregnant or breastfeeding<br>4. Participation in a clinical trial of an investigational medicinal product (other than antibiotics or anti-virals) within 30 days<br>5. Major trauma in the prior 5 days<br>6. Presence of any active malignancy (other than non-melanoma skin cancer) that required treatment within the last year<br>7. WHO Class III or IV pulmonary hypertension<br>8. Pulmonary embolism within past 3 months<br>9. Currently receiving extracorporeal life support (ECLS)<br>10. Chronic kidney disease receiving dialysis

11. Severe chronic liver disease with Child-Pugh score > 12
12. DNAR (Do Not Attempt Resuscitation) order in place
13. Treatment withdrawal imminent within 24 hours
14. Prisoners
15. Non-English speaking patients or those who do not adequately understand verbal or written information unless an interpreter is available.
16. Enrolled in a concomitant clinical trial of a medicinal product (other than antibiotics or anti-virals)
17. IgA deficiency

Schematic of Study Design:

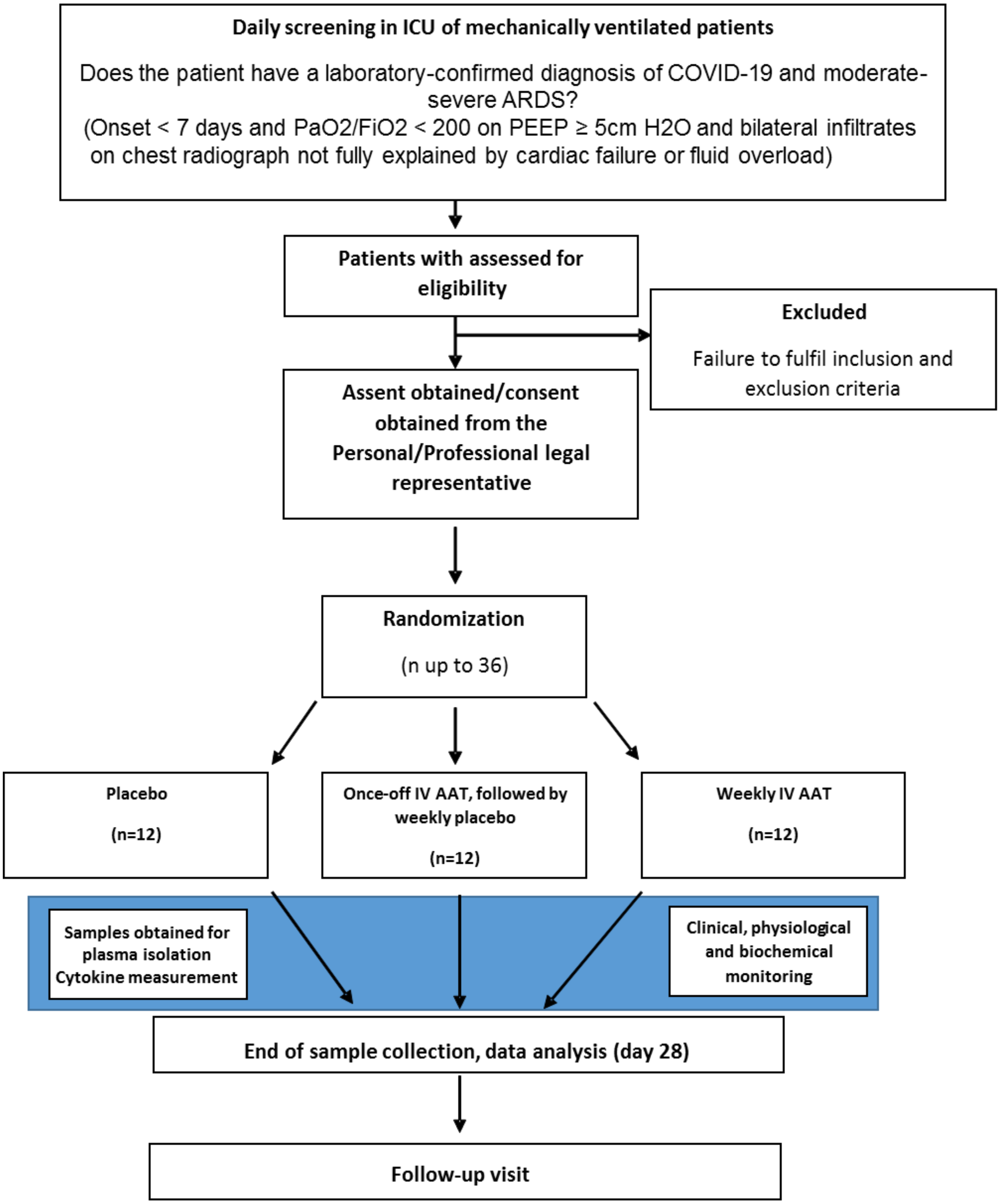
Daily screening in ICU of mechanically ventilated patients
Does the patient have a laboratory-confirmed diagnosis of COVID-19 and moderate-severe ARDS?
(Onset < 7 days and PaO2/FiO2 < 200 on PEEP ≥ 5cm H2O and bilateral infiltrates on chest radiograph not fully explained by cardiac failure or fluid overload)
Patients with assessed for eligibility
Excluded
Failure to fulfil inclusion and exclusion criteria
Assent obtained/consent obtained from the Personal/Professional legal representative
Randomization
(n up to 36)
Placebo
(n=12)
Once-off IV AAT, followed by weekly placebo
(n=12)
Weekly IV AAT
(n=12)
Samples obtained for plasma isolation Cytokine measurement
Clinical, physiological and biochemical monitoring
End of sample collection, data analysis (day 28)
Follow-up visit

8. Key Roles and Contact Information

Chief Investigator:	Prof. Ger Curley (MB, BCh, BAO (Hons), MSc (Hons), PhD, FCARCSI, FJFICMI)<br><br>Professor of Anaesthesia and Critical care, Beaumont Hospital, Beaumont Rd, Dublin 9, Ireland. Tel: : +353 1 8093810 Fax: +353 1 8093755 Email: gercurley@rcsi.ie
Clinical Site Investigators:	Prof Ger Curley, Beaumont Hospital, Dublin, Ireland.<br><br>Prof Noel G. McElvaney, Beaumont Hospital, Dublin, Ireland.<br><br>Prof John Laffey<br>Professor of Anaesthesia and Intensive Care Medicine<br>NUI Galway<br>Tel: +353-91-495662<br>Email: john.laffey@nuigalway.ie<br><br>Dr Ignacio Martin Loeches<br>Vice-Chair of Intensive Care Medicine<br>St James's University Hospital, Dublin<br>Email: imartinl@tcd.ie<br><br>Dr Brian Marsh<br>Consultant in Anaesthesia & Intensive Care Medicine<br>Mater Misericordiae University Hospital<br>Email: bmarsh@mater.ie
Institutions:	Intensive Care Unit, Beaumont Hospital Beaumont Rd, Dublin 9, Ireland Prof. Ger Curley Tel: : +353 1 8093810 Fax: +353 1 8093755 Email: gercurley@rcsi.ie<br><br>Respiratory Research Laboratory, RCSI Smurfit Building, Beaumont Hospital,Dublin 9, Ireland Prof. Noel G McElvaney Tel: : +353 1 8093763 Fax: +353 1 8093765 Email: gmcelvaney@rcsi.ie<br><br>Department of Anaesthesia and Critical Care, RCSI Smurfit Building, Dublin 9, Ireland Prof. Ger Curley Tel: : +353 1 8093810 Fax: +353 1 8093755 Email: gercurley@rcsi.ie<br><br>Department of Medicine, RCSI Smurfit Building, Beaumont Hospital,Dublin 9, Ireland Prof. Noel G

	McElvaney Tel: : +353 1 8093763 Fax: +353 1 8093765 Email: gmcelvaney@rcsi.ie<br><br>Department of Anaesthesia and Intensive Care Medicine, NUI Galway. Prof John Laffey. Tel: +353-91-495662. Email: john.laffey@nuigalway.ie<br><br>Department of Intensive Care Medicine, St James's University Hospital, Dublin. Dr Ignacio Martin Loeches. Email: imartinl@tcd.ie<br><br>Department of Anaesthesia & Intensive Care Medicine, Mater Misericordiae University Hospital, Dublin. Dr Brian Marsh. Email: bmarsh@mater.ie
Statistician	Fiona Boland, Royal College of Surgeons in Ireland Data Science Centre. Tel: +35314022507. Email: fboland@rcsi.ie

9. Introduction

9.1 Background

COVID-19

In early December 2019, multiple pneumonia cases of unknown origin were identified in Wuhan, the capital city of Hubei province, China. The inciting pathogen was subsequently identified as a novel enveloped RNA betacoronavirus, now known as severe acute respiratory syndrome coronavirus 2 (SARS-CoV-2), or, colloquially, COVID-19. The World Health Organization (WHO) has recently declared COVID-19 a public health emergency of international concern. As of March 30, 2020, more than 750,000 laboratory-confirmed cases have been documented globally, with over 36,000 deaths. In Ireland, there have been over 2,800 confirmed cases to date, with 46 deaths. These numbers are expected to grow substantially in the coming weeks, as testing becomes more widely available and a critical mass of infected individuals leads to patient overflow from hospitals into the community.

Early data from clinical and research facilities in both China and Italy indicate that following the introduction of formalized testing programmes, the number of diagnosed cases rises exponentially. This in turn results in bed capacity in healthcare facilities being exceeded. Availability of critical care medicine (intensive care unit, critical care unit, coronary care unit) is limited by a scarcity of ventilators, invasive monitoring equipment, specialist staff and critical care beds. The projected increase in diagnosed cases in Ireland is 30% per day, with an estimate of 10-15,000 cases by the end of this month (March). The reported proportion of patients unwell enough to require ICU support is 6-14%. The median time spent on a ventilator by an infected patient requiring ICU support is 7 days. Ireland currently has less than 300 ICU beds.

ARDS

ARDS is a common condition of acute hypoxemic respiratory failure resulting from disruption of the alveolar-capillary barrier, and a resultant inflammatory pulmonary oedema. It can occur in the critically ill in response to many insults including severe trauma, burns, infection, major surgery or neurological disorders. ARDS is a leading cause of death and disability in critically ill adults and children worldwide 1, with a hospital mortality of approximately 40%. It is estimated that up to 20,000 cases of ARDS occur annually; 50% of these fall into the “moderate -severe” category of ARDS and have up to 40% mortality. ARDS confers a considerable long-term illness and disability burden on the individual sufferer and on society. Only 50% of survivors are able to return to work 12 months after hospital discharge, while cognitive, psychologic, and physical morbidity persists for up to 5 years.

ARDS has significant resource implications, prolonging intensive care unit (ICU) and hospital stay, and requiring rehabilitation in the community, all of which stand to be impaired by the re-deployment of resources during the current pandemic. Prior to the emergence of COVID-19, patients with ARDS accounted for 10% of intensive care unit (ICU) admissions, and over a

quarter of ICU bed-days. The rapid accumulation of COVID-associated ARDS cases both in Ireland and internationally will therefore lead to ICU capacity being exceeded.

There are no specific treatments for ARDS, and management remains supportive with lung-protective ventilation and restrictive fluid management 8. Among patients who receive low tidal–volume ventilation, mortality rates remain unchanged over the past two decades highlighting the need for novel therapeutic strategies. Unsuccessful large scale clinical trials of multiple therapeutic strategies, including nitric oxide, anti-oxidants, surfactants, corticosteroids and immunomodulating agents such as IL-10 , GM-CSF, neutrophil elastase inhibitors, and high frequency oscillatory ventilation highlight the need for novel approaches for these patients. Most recently, statin therapy was found to be ineffective in 2 large trials, including the HARP-2 study of simvastatin. Therefore, innovative therapies are needed to reduce both the mortality associated with ARDS and the long-term morbidity in survivors.

Proposed trial population

The trial population we propose to study includes any patient admitted to the ICU with moderate-to-severe ARDS secondary to COVID-19 ($PaO_2/FiO_2 <200$) Patients will where possible be matched for age.

Proposed intervention

The intervention we propose to use is human plasma-purified alpha-1 antitrypsin (Prolastin, Grifols Pharmaceuticals, Germany). This medication has previously been administered to humans intravenously on a weekly basis to patients with alpha-1 antitrypsin (AAT) deficiency, graft-versus-host disease and severe panniculitis [1-5]. We propose to use a dose of 120mg/kg. This medication has an established safety profile at this dose (and also for more frequent administration) both in the clinical trial setting and in clinical practice.

Overall aim of the study

Briefly, the study aims to assess whether treatment with Prolastin results in decreased levels of pro-inflammatory cytokines, decreased inflammation and improved outcome in patients with COVID-19-associated ARDS.

Clinical, epidemiological, and public health background of COVID-19; this study in context

Little is known about the pre-hospital course of the disease. However, in-hospital studies have shown that COVID-19 induces excessive and aberrant non-effective host immune responses associated with severe lung injury [6]. Higher rates of ARDS are seen in elderly patients with comorbidities [7]. In China, although 80% had mild illness, approximately twenty five percent of those hospitalized needed ICU care, and for those in the ICU, mortality was 49%. Those with Acute Respiratory Distress Syndrome (ARDS) have a mortality of 52.4% [8, 9].

In those with COVID-19 infection and ARDS, severe systemic inflammation is also observed, with several key inflammatory cytokines the likely culprits, in particular interleukin (IL)-6 and IL-1β. Onset of this highly-proinflammatory state has been noted in several of the above

studies to occur prior to rapid clinical deterioration in the context of acute respiratory failure, ARDS, acute renal failure, shock and arrhythmia, all of which have are known to be related to upregulation of one or more of the above-mentioned cytokines. The risk of dying appears highest in those with older age, hypertension, diabetes and lower temperature.

No antiviral or other drug has shown clinical efficacy in treating these individuals. Therapeutic options that have been proposed include steroids, intravenous immunoglobulin and selective cytokine blockade targeting two master pro-inflammatory cytokines involved in viral illness and ARDS - namely IL-1β and IL-6 - such as anakinra and tocilizumab [10]. Clinical trials with the combination lopinavir/ritonavir, remdesivir and tocilizumab are still ongoing. One recent randomized evaluation of lopinavir/ritonavir in 199 patients in China showed no benefit compared to placebo [11]. The evidence for the potential effectiveness of chloroquine is based on in vitro data and unpublished single arm studies in China, but nevertheless many centres are now using hydroxychloroquine on an off-label basis [10]. In one single arm study on 20 confirmed COVID-19 patients, treatment with hydroxychloroquine was associated with viral load reduction and this effect seemed to be reinforced by the combination with azithromycin [10].

Such empiric therapy conveys clear potential safety risks, therefore the priority should be recruitment of patients into well-designed clinical trials to ensure the assessment of safety and effectiveness of treatment options in addition to biological efficacy before proceeding to larger multi-centre studies.

Importance of the study

If the proposed intervention is successful, it would represent the first successful randomized-placebo control trial in the field and the first clinically-proven treatment for severe COVID-19 illness beyond standard ICU management of ARDS. This would facilitate progression to a larger multi-centre study. The number of lives saved in the event of such a therapy proving efficacious with regard to clinical outcomes would conservatively stand in the hundreds of thousands, and potentially in the millions.

9.2 Preclinical data

One of the primary functions of AAT in the human body is to irreversibly bind neutrophil elastase (NE) an omnivorous serine protease release by activated or disintegrating neutrophils. NE levels and activity are markedly increased in the airways of patients with ARDS, and previous studies have shown that IV administration of AAT in humans results in augmented antielastase capacity of airway epithelial lining fluid, reduced NE activity in bronchoalveloar lavage fluid and decreased breakdown of lung tissue [1, 2, 4, 5]. While AAT chiefly inhibits NE, it also other exerts an antiprotease effect on chymotrypsin, cathepsin G (CathG) and proteinase 3 (PR3) [12] . The structure of the AAT is critical for its antiprotease activity and comprises 3 beta sheets (A, B and C), 9 alpha helices and a reactive center loop (RCL) at the C-terminal end [13].

In addition to its antiprotease effects [14-16], AAT is a potent anti-inflammatory and immunomodulator [17-21]. Of particular relevance to COVID-19, it has been shown to modulate suppress the production and activity of several key pro-inflammatory cytokines, including interleukin IL-1, IL-6, IL-8 and tumor necrosis factor (TNF)-α [17, 18, 22, 23], while preserving the production of the anti-inflammatory cytokine IL-10 [24]. AAT also binds a variety of proteins and fatty acids [25, 26].

Research investigating the impact of AAT on viral infections has demonstrated that HIV replication and T-cell numbers are associated with low serum levels of AAT and recent data indicate that AAT can inhibit HIV replication in T-cells as quantified by significant decreases in viral RNA and p24 epitope. This latter observation is thought in part due to the ability of AAT to enter T cell and impact upon IjBa ubiquitination [27]. A peptide derived from AAT has also been shown to display antiviral properties in response to HIV-1 infections – Munch, et al., [28] showed that the AAT viral inhibitory peptide (VIRIP) inhibited a variety of HIV-1 strains including those resistant to current antiretroviral drugs and demonstrated that VIRIP blocked HIV-1 entry by interacting with the gp41 fusion peptide.

9.3 Clinical data

Plasma-purified AAT has been administered intravenously to patients with AATD since the 1980's in order to achieve plasma levels exceeding a protective threshold of 11µM [5]. The initial dose was 60mg/kg given once weekly but other doses trialled following that including 120mg/kg biweekly [29] and 250mg/kg every 28 days [30]. Each dosing regimen was safe and well tolerated. Plasma purified AAT (usually at a dose of 60mg/kg) has been shown to impair destruction of lung tissue and slow progression of emphysema in people with severe AAT-deficiency [2, 4].

Patient outcomes from two major studies of patients following AAT augmentation therapy suggest that it appears well tolerated and is generally safe [31, 32]. Wencker, et al., [32] reported the trial of 443 augmentation therapy recipients, of whom 65 experienced a total of 124 adverse events. Most commonly encountered adverse reactions were relatively benign and self-resolving. These included chills, urticarial rashes, fatigue, nausea, and vomiting. Although specific adverse effects may be anticipated and specifically monitored for during therapy, the range of possible adverse effects is large, and new potentially unforeseeable effects remain possible. The trial data however are reassuring, given that 58,000 infusions resulted in only five severe adverse events (four patients suffered anaphylactic reactions and one patient developed congestive cardiac failure), all of which concluded with a complete recovery. Critically, no deaths or instances of hematologic viral transmission were observed.

Stoller et al [31] reported on the NHLBI experience of 747 patients receiving weekly AAT augmentation therapy for a period of seven years. The overall rate of adverse events was low at 0.02 per patient-month, with 83% of patients reporting no events. There were a total of 720 recorded adverse events, the most common of which included headache (47%), dizziness (17%), nausea (9%), and dyspnoea (9%).

More recent data has shown that increasing the dosage of plasma purified AAT given to AATD patients not only increases the anti-protease protection but also significantly decreases inflammation across a wide spectrum of inflammatory pathways including many of the cytokines seen in critically ill patients with COVID-19 [1]. AAT is also known to be an endogenous inhibitor of many pro-inflammatory cytokines associated with COVID-19 infection including interleukin-6, IL-1β and TNF-α. Indeed, we have recently shown that abrupt cessation of AAT augmentation therapy for patients with genetic AAT-deficiency results in alterations to immune cell metabolism, inflammasome activation, marked increases in levels of these specific aforementioned pro-inflammatory cytokines, and subsequent progression to respiratory failure (see supplementary appendix).

Further systemic effects of high dose AAT administration include a dramatic effect in the treatment of panniculitis [33, 34]. In addition there are circumstances when AAT is present in the body in normal amounts but is inactivated, either by excess inflammation leading to oxidation, complexation or proteolytic cleavage [35].

9.4 Rationale and risks/benefits

The study is designed so that:

- One out of 3 patients will receive placebo once weekly for 4 weeks,
- One out of 3 patients will receive a single dose of plasma purified AAT at a dose of 120mg/kg of body weight via intravenous infusion followed by placebo once weekly for 3 weeks, and
- One out of 3 patients will receive a once weekly dose of plasma purified AAT at a dose of 120mg/Kg once weekly for 4 weeks.

This will be followed by an observational week for all study participants.

The medication will be given intravenously as there are compelling data to show that AAT has systemic and lung anti-inflammatory effects [25, 26, 31, 34].

The dose of 120mg/kg was chosen given the half-life of AAT following intravenous administration which is 4.6 days. In the study of Campos, et al., [1] a 120mg/kg infusion resulted in an increase in AAT level by 22µM at the nadir just before the next infusion which in non-AAT deficient individuals would lead to an AAT level in plasma between 42-75µM at day 7 post infusion. The data we have from healthy control individuals with community acquired pneumonia is that their AAT level increases to 53 +/- 18µM on acute presentation and falls to 28.4 +/- 8.1µM at 10 days. In data generated from our ICU from patients with ARDS, those who died had a significantly lower AAT level than those who survived (please see supplementary appendix). This dose of 120mg/kg has been shown to decrease pro-inflammatory cytokines in peripheral blood and in the lungs [1]. In addition a similar dose regimen has been sued in refractory panniculitis to good effect.

The study population chosen will be the most severely ill patients with COVID-19 for whom at the moment only supportive therapy is available. This group has acute respiratory distress syndrome (ARDS) and a mortality of >50% [8, 9].

Our hypothesis is that there is a systemic and local deficiency of active AAT in people with ARDS and specifically people with ARDS secondary to COVID-19. This condition is characterized by severe cytokinaemia particularly with IL-6. AAT is an inhibitor of IL-6 production in addition to its effects on other cytokines. AAT also expresses antiviral properties and inhibits many of the pro-inflammatory actions of those cytokines with particular emphasis on neutrophils and T-cells two for the major cell types thought to be central to the lung manifestations of COVID-19.

The currently available treatments for COVID-19 associated ARDS are supportive. A variety of approaches have been tried but no antiviral or other specific medication is currently recommended. Therapeutic options that have been proposed, include steroids, intravenous immunoglobulin, selective cytokine blockade (e.g. anakinra or tocilizumab) and JAK pathway inhibition. Clinical trials with the combination lopinavir/ritonavir, remdesivir and tocilizumab are ongoing, but to date are not convincing. The evidence for the potential effectiveness of chloroquine is based on in vitro data and unpublished single arm studies in China. In contrast, AAT is a proven anti-inflammatory against many of the processes associated with COVID-19 lung disease.

9.5 Assessment and management of risk

Risk/benefit

AAT has been extensively used in individuals with severe lung disease for the past 40 years. It has an exceptional safety record. In two major studies of patients with deficiency of AAT undergoing chronic AAT augmentation therapy, the therapy was shown to be well tolerated and generally safe [31, 32]. As stated in 9.3 above, Wencker, et al., [32] reported the trial of 443 augmentation therapy recipients, of whom 65 experienced a total of 124 adverse events. Most commonly encountered adverse reactions were relatively benign and self-resolving. These included chills, urticarial rashes, fatigue, nausea, and vomiting. Although specific adverse effects may be anticipated and specifically monitored for during therapy, the range of possible adverse effects is large, and new potentially unforeseeable effects remain possible. The trial data however are reassuring, given that 58,000 infusions resulted in only five severe adverse events (four patients suffered anaphylactic reactions and one patient developed congestive cardiac failure), all of which concluded with a complete recovery. Critically, no deaths or instances of hematologic viral transmission were observed.

Stoller et al [31] reported on the NHLBI experience of 747 patients receiving weekly AAT augmentation therapy for a period of seven years. The overall rate of adverse events was low at 0.02 per patient-month, with 83% of patients reporting no events. There were a total of 720 recorded adverse events, the most common of which included headache (47%), dizziness (17%), nausea (9%), and dyspnoea (9%). It is important to note however that the

data highlighted a lower rate of adverse events in participants receiving augmentation therapy less frequently than weekly. The rate of total adverse events decreased from 0.03 events per patient- month at weekly infusions to 0.024 events per patient-month at 2–3 weeks (P=0.020) and 0.005 events per patient-month at monthly infusions. Meyer et al (22) reported a very rare IgE anaphylactic reaction following the third intravenous infusion of AAT. Specific IgE antibodies against Prolastin-HS were identified on serum analysis of the patient. A second case has yet to be described in the literature.

Although the usual dosage is 60mg/kg, recent large scale studies have evaluated safety in those receiving 120mg/Kg dosing. The largest of these studies evaluated the RAPID-RCT and RAPID-OLE studies in which biweekly infusions comprised 4.3% of all AAT and 5.2% of all placebo administrations across both studies [29]. The calculated drug exposure for 60 m/kg infusions weekly was >10 times higher than for the biweekly 120 mg/kg infusions (AAT 158.20 versus 14.09 subject-years, placebo 134.92 versus 15.55 subject-years). When all AAT biweekly doses were pooled across RAPID-RCT and -OLE, 933 infusions over 884 infusion sequences in 137 patients were identified (mean±sd 6.45±4.26, range 1–21).

The exposure adjusted event rates (EARS) were 7.32 versus 6.08 for weekly 60 mg/kg AAT and placebo (p=0.103) and 7.45 versus 7.08 for biweekly 120 mg/kg AAT and placebo infusions (p=0.754), respectively. The proportion of patients who experienced a treatment emergent adverse event (TEAE) during the infusion sequences was 33.3% in the biweekly 120 mg·kg−1 group compared to 35.7% in biweekly placebo and 37.3% in weekly 60 mg/kg or 37.1% in weekly placebo. The corresponding infusion adjusted event rates (IAER) were 0.1682 versus 0.1386 for 120 mg/kg AAT and placebo infusions (p=0.754) and 0.1464 versus 0.1265 for 60 mg/kg AAT versus weekly placebo (p=0.103). Within 24 h of infusion, when volume-related TEAEs would be expected, IAERs were 0.0374 for 120 mg/kg AAT versus 0.0301 for biweekly placebo, and 0.0343 for mg/kg AAT versus 0.0361 for weekly placebo. IAERs in the delayed-start OLE subgroup were 0.0645 in the 60 mg compared to 0.1111 in the 120 mg periods (p=0.0386). During all double-dose infusion sequences four TEAEs were reported in association with the biweekly 120 mg/kg AAT infusions (one patient reported joint pain and headache, one had an exacerbation and one complained of headache; cumulative IAER=0.0125), compared to a single treatment-related report of rash in the placebo biweekly regimen (IAER=0.0030).

During RAPID-RCT and -OLE 16% of patients reported a TEAE within 24 h of infusion and 25% reported TEAEs within 72 h in either treatment group. A higher proportion of patients experienced a TEAE within 7 days following a biweekly 120 mg/kg AAT infusion (43.8%) than 7 days prior to double-dosing (34.3%). The number of TEAEs per infusion was 0.1267 for 120 mg/kg AAT 1 and 0.1041 for 60 mg/kg AAT. Most TEAEs were mild or moderate in intensity: 94.6% with mg/kg AAT and 92.9% with biweekly 120 mg/kg AAT. An analysis of TEAEs by system organ class showed similar distributions in all subgroups.

In RAPID-RCT four SAEs were reported during the infusion sequences three SAEs with biweekly 120 mg/kg AAT (malignant tumour in the bladder, transurethral resection of the prostate and small bowel obstruction); one SAE was reported with mg/kg AAT (chest pain); and none were reported with placebo. In RAPID-OLE eight SAEs occurred (exacerbations n=4; three adverse events (pneumonia, abscess and chronic carnification of the lobe) in a

single patient and brain thrombus n=1). One of the 12 SAEs was considered to be drug related.

The IMP may achieve clinical improvement in this patient cohort as augmenting the body's anti-inflammatory, antiprotease defenses with AAT may help the body withstand the enormous inflammatory attack which overwhelms the normal AAT response. The higher dosage is justified given our knowledge of the pharmacokinetics of plasma purified AAT, the need to achieve high levels in the circulation and lung and the published data on the enhanced anti-inflammatory effects with higher doses. We do not know the duration of treatment required. We have some data that a single high -dose of AAT may achieve the desired response but given the violent nature of the physiologic disturbances in COVID-19 ARDs we think it prudent to also include a more chronic dosing regimen. There is a possibility that by the time the patient is intubated with COVID-19 related ARDs we will have missed the opportunity to significantly interrupt an inflammatory process, which has begun and will continue unabated and that earlier intervention might be warranted.

There is no evidence that the COVID-19 population will handle AAT differently than other populations but there is no COVID-19 specific data in that regard. Plasma purified AAT has frequently been administered in the presence of significant liver disease (another common manifestation of AAT deficiency) with no harmful side effects. It has also been administered to elderly patients with no need for dose adjustment and to immunocompromised individuals. There have been no specific studies of AAT administration in people with severe renal failure, although it is not a contraindication to its use.

AAT is frequently administered to people with significant lung and liver disease who are on many concomitant medications. There are no known interactions.

The risk for administering AAT to individuals with ARDS in the setting of COVID-19 is not a significant risk. This medication has a very high safety profile and will be administered in an institution with a very high expertise in this area. The medication is administered over approximately 60 minutes in a low volume. There is no specific training involved in its administration. It will be reconstituted by unblinded trial personnel just prior to administration.

The potential benefits of this medication in this context is to see a decrease in the cytokinemia in these patients, with reversal or amelioration of the hemodynamic disturbances following from that.

9.6 Potential Risks

Prolastin is currently used worldwide, most notably for the treatment of people with genetic AAT-deficiency. Other similar preparations for augmentation include Aralast/Glassia (Baxter), and Zemira (CSL Behring), all of which are purified protein intravenous formulations taken from pooled human plasma.

While Prolastin has been given to humans successfully for over 40 years, and has an excellent safety profile, it is important to consider potential risks, however unlikely, pertaining to its use.

Risks of Prolastin:

- Because Prolastin is made from human blood, it may carry the risk of transmitting infectious agents. The risk that such products will transmit an infectious agent has been reduced by screening plasma donors for prior exposure to certain viruses, by testing for the presence of certain current virus infections, and by inactivating and/or removing certain viruses.
- There is also the possibility that unknown infectious agents may be present in such products
- There will be an increase in plasma volume following intravenous administration of Prolastin. Caution should therefore be used in patients at risk for circulatory overload
- Adverse drug reactions are uncommon with Prolastin, both in the clinical trial and clinical practice setting
    - In clinical studies, 6 reactions were observed with 517 infusions of Prolastin, or 1.16%. None of the reactions were severe. The adverse reactions reported included delayed fever (maximum temperature rise was 38.9°C, resolving spontaneously over 24 hours) occurring up to 12 hours following treatment (0.77%), lightheadedness (0.19%), and dizziness (0.19%). Mild transient leukocytosis and dilutional anemia several hours after infusion have also been noted.
    - Additionally, since market entry of Prolastin, occasional reports of the following events have been received: flu-like symptoms, allergic-like reactions, dyspnea, tachycardia, shortness of breath, bronchospasm, wheezing, urticaria, back pain, clamminess, sweating, diarrhea, and fatigue. Less frequently, the following have also been reported: hypotension, anxiety, cyanosis, swelling of hands and feet, angio-, facial and lip oedema, nasal congestion, sinusitis, abdominal pains or cramps, pallor, and weakness. Rare cases of transient increase in blood pressure or hypertension and chest pain have also been reported.
    - Given that the proposed use of Prolastin in this instance is off-label, a risk/benefit analysis has been conducted in advance of this application. The analysis concluded that the grave need for therapeutics in this critically ill cohort, combined with the excellent real-world safety profile of Prolastin, its predictable pharmacokinetics and pharmacodynamics, its clearly delineated dosage and route of administration, the pronounced anti-inflammatory effect demonstrated in previous off-label uses, and the biological plausibility of AAT as a means of reducing the inflammatory burden in these patients, outweighed the low likelihood of adverse events.

9.7 Potential Benefits

It is hoped that participants receiving Prolastin will experience decreased inflammation and clinical improvement. In the longer term, scientific and medical knowledge gained in this study may enhance our understanding of the disease process and lead to much-needed treatments for severe COVID-19-associated illness, as described in 9.1 above.

10 Objectives

10.1 **Primary Objective(s)**

The aim of the study is to conduct a clinical trial of IV AAT as a prospective anti-inflammatory therapy for severely ill COVID-19 patients with ARDS requiring ICU admission.

The primary objective is to demonstrate a biological effect of IV Prolastin administered weekly at 120mg per kilogram of body weight in patients with severe COVID-19 illness requiring intubation and mechanical ventilation for ARDS by reducing circulating levels of IL-6 as measured by plasma ELISA.

10.2 **Secondary Objective(s)**

The secondary objectives of this study are:

1. To determine the safety and tolerability of [IV Prolastin administered once at 120mg per kilogram of body weight] and [IV Prolastin administered weekly at 120mg per kilogram of body weight for 4 weeks], as assessed by the number of AEs and SAEs.

and to determine the effects of [IV Prolastin administered once at 120mg per kilogram of body weight] and [IV Prolastin administered weekly at 120mg per kilogram of body weight for 4 weeks] on:

2. Physiological indices of respiratory dysfunction reflecting severity of ARDS, as measured by oxygenation index (OI) and respiratory compliance

3. Sequential organ failure assessment (SOFA) score, an assessment of clinical severity in critically unwell patients

4. Mortality, given that no treatment to date has been shown to influence this outcome

5. Time on ventilator, given the impending shortage of ICU bed availability

6. Circulating alpha-1 antitrypsin (AAT) levels

7. Circulating levels of IL-1β, IL-8, IL-10, soluble TNF receptor 1 (a surrogate marker for TNF-α), all of which have been implicated in pulmonary and systemic inflammation, and also to be suppressed by AAT in vivo

8. Development of shock, a key cause of death in this cohort

9. Acute kidney injury, a key cause of death in this cohort

10. Need for renal replacement therapy, given the impending shortage of renal replacement facilities for the population studied

11. Clinical relapse, since this must be clarified to facilitate safer discharge from an ICU setting.

12. Length of ICU stay in days

10.3 Study Outcome Measures

10.3.1 Primary outcome measure

The primary outcome to be assessed is the change in level of circulating IL-6 in plasma at 7 days as measured by ELISA.

10.3.2 Secondary outcome measure

Secondary endpoints for this study are listed below. These endpoints are designed to inform a larger multi-centre study in the future, should biological anti-inflammatory efficacy be demonstrated in the present study. Larger number of participants would likely be required to detect an effect on many of these secondary endpoints. However, should the present study detect an effect of Prolastin therapy one one of the secondary endpoints, such a result would be of great importance to the international medical community.

Secondary outcome measures:

• Safety and tolerability of both once-off and weekly administration of IV AAT in the cohort

• PaO2/FiO2 ratio

• Respiratory compliance

• Sequential organ failure assessment (SOFA) score

• Mortality

• Time on ventilator in days

• Circulating AAT levels as measured by nephelometry

• Plasma levels of IL-1β as measured by ELISA

• Plasma levels of IL-8 as measured by ELISA

• Plasma levels of IL-10 as measured by ELISA

• Plasma levels levels of soluble TNF receptor 1 (sTNFR1, a surrogate marker for TNF-α) as measured by ELISA

• Development of shock, defined for the purpose of this study as life-threatening organ dysfunction caused by a dysregulated response to infection, with critical reduction in tissue perfusion and acute failure of multiple organs, including the lungs, kidneys, and liver

• Acute kidney injury defined as an abrupt sustained rise in urea and creatinine

• Need for renal replacement therapy

• Clinical relapse, as defined by the need for readmission to the ICU or a marked decline in PaO2/FiO2 or development of shock or mortality following a period of sustained clinical improvement

• Secondary bacterial pneumonia as defined by the combination of radiographic findings and sputum/airway secretion microscopy and culture.

• Length of ICU stay

• Need for, and duration of, vasopressor therapy

11 Trial design

11.1 Overall design

Purpose of research: non-commercial trial

Type/design of trial to be conducted: a randomized, placebo-controlled, pilot, double blind parallel group study of [IV Prolastin administered once at 120mg per kilogram of body weight] versus [IV Prolastin administered weekly at 120mg per kilogram of body weight for 4 weeks] versus placebo. Both the active drug and matching placebo (0.9% sodium chloride solution for infusion) will be prepared by unblinded trial personnel and then transferred to the ICU in a blinded fashion to be administered at the bedside by a blinded member of the trial team. This process will be further detailed in a trial specific SOP for IMP preparation and administration.

Description of the study population: The trial population we propose to study is a critically unwell population, and includes any patient admitted to the ICU with moderate-to-severe ARDS secondary to COVID-19 (PaO2/FiO2 <200). Patients will where possible be matched for age.

Rationale for design features: The comparison of [IV Prolastin administered once at 120mg per kilogram of body weight] versus placebo will indicate whether Prolastin exerts an anti-inflammatory effect in this population, and whether it is safe and well-tolerated. The comparison of [IV Prolastin administered once at 120mg per kilogram of body weight] versus [IV Prolastin administered weekly at 120mg per kilogram of body weight for 4 weeks] will clarify whether (1) a clinical deterioration is merely delayed by a single dose of Prolastin,

rather than abolished entirely, (2) the anti-inflammatory effect is more pronounced with repeated dosing, and (3) once-off dosing and repeated weekly dosing are equally safe.

Phase of trial: This study constitutes a single-centre Phase 2 trial that will inform a larger multi-centre Phase 3 trial.

The number of study groups/arms: There are 3 study groups

Description of study groups/arms including sample size: Group 1 (n=12) will receive placebo once weekly for 4 weeks. Group 2 (n=12) will receive IV Prolastin at 120mg/kg once, and following 7 days will then receive placebo weekly for a further 3 weeks (a total of 4 weeks of AAT or placebo). Group 3 (n=12) will receive IV AAT at 120mg/kg weekly for 4 weeks

Time to complete study enrolment: The projected time required to complete study enrolment is 12 months.

Expected duration of subject participation: The expected duration of subject participation is 5 weeks, with the first 4 weeks involving patients receiving either drug or placebo. This will likely involve patients continuing to be involved in the study beyond their discharge from ICU. This is intentional, and is designed to assess these individuals for delayed decline, a key feature of the mortality associated with COVID-19 illness. Following completion of the study, patients will subsequently be offered follow-up in a dedicated post-COVID-19 clinic.

Identification and specifics of administration of the study intervention: The intervention we propose to use is human plasma-purified alpha-1 antitrypsin (Prolastin, Grifols Pharmaceuticals, Germany). This medication is administered intravenously on a weekly basis to patients with alpha-1 antitrypsin (AAT) deficiency, graft-versus-host disease and severe panniculitis. We propose to use a dose of 120mg/kg. This medication has an established safety profile at this dose (and also for more frequent administration) both in the clinical trial setting and in clinical practice. The placebo will be 0.9% sodium chloride for infusion. Patients will continue to receive current standard of care treatment during the study. Both patients and clinical staff administering the drug/placebo will be unaware as to whether drug or placebo is being administered. Study investigators conducting lab-based experimental measurements will be unaware as to whether the samples they are performing measurements in are from patients who are receiving drug or those receiving placebo. Similarly they will be unaware of the clinical status or trajectories of these patients.

Sequence and duration of all trial periods: The 3 groups will run in parallel. The study duration for each group is 5 weeks, beginning with 4 weeks of drug or placebo and ending with a week of no drug/placebo. Patients will continue to receive follow-up from the clinical team, and will also be offered follow-up via a dedicated post-COVID-19 follow-up clinic.

Planned variation in intervention dose or schedule: There is no dose escalation element to this study. Patients will receive either [IV Prolastin administered once at 120mg per kilogram of body weight] or [IV Prolastin administered weekly at 120mg per kilogram of body weight for 4 weeks] or [IV placebo administered weekly for 4 weeks].

Methods for collecting data for assessment of study objectives: Clinical evaluation will be by the COVID team on-call in conjunction with the ICU consultant on call. Patients will be

enrolled if they satisfy inclusion/exclusion criteria. Demographic and clinical information will be collected from the medical chart record and pseudonymised before being transferred to a password protected encrypted spreadsheet stored on a password protected computer. The clinical data pertaining to a given patient will already be accessible to the patient's designated health care team. The overall clinical data set will be accessible to the study data controller, and to designated data processors. Blood will be taken from the patient during routine blood draws from an in-situ arterial line by the clinical team assigned to their care. Results of hospital blood tests undertaken as part of standard care will be monitored. At two-day intervals, an extra 10-15ml of blood will be taken for the purpose of plasma isolation. This entails centrifuging whole blood in lithium heparin tubes at 200 x g for 5minutes at room temperature and removing the plasma supernatant that results for storage at -80oC. Cytokine measurements (IL-1, IL-6, IL-8, sTNFR1 and IL-10) will be undertaken using ELISA in accordance with manufacturers instructions. Experiments will be conducted under BSL2 conditions by operators using personal protective equipment (PPE). Physiological parameters such as respiratory compliance and PaO2/FiO2 will be monitored as part of routine clinical care. Clinical outcomes (mortality, time on ventilator, shock, renal replacement therapy) will be recorded on a time-to-event basis. All biological and physiological results, as well as clinical outcomes, will be stored on a password protected encrypted spreadsheet, which is in turn stored on a secure server hosted by the data controller.

Other protocol-specific details: For this study, a central hospital laboratory will be used for measurements undertaken as part of routine clinical care, a central treatment area with specialist staff will be used, the investigational products (drug/placebo) will be made up in a centralized unit by a trained practitioner, the statistical analysis will be undertaken by a neutral statistician not connected with the study team, and cytokine measurements and isoelectric focusing will be undertaken by blinded research scientists in a centralized research laboratory.

11.2 Sub-studies (if applicable)

Not applicable.

12 Selection of Subjects

12.1 Inclusion criteria

In order to be eligible to participate in this study, an individual must meet all of the following criteria:

1. Laboratory confirmed diagnosis of COVID-19 infection.

2. Moderate to severe ARDS with a PaO2/FiO2 ratio <200

3. >18 years of age

4. Patients receiving invasive mechanical ventilation or non-invasive ventilation

Fulfilment of each criterion must be clearly evidenced (in lab reports or correspondence) and/or documented in the medical records.

12.2 Exclusion criteria

All individuals meeting any of these exclusion criteria at baseline or during screening will be excluded from study participation:

1. More than 96 hours from the onset of ARDS
2. Age < 18 years
3. Known to be pregnant or breastfeeding
4. Participation in a clinical trial of an investigational medicinal product within 30 days (other than antibiotics or anti-virals)
5. Major trauma in the prior 5 days
6. Presence of any active malignancy (other than non-melanoma skin cancer) that required treatment within the last year
7. WHO Class III or IV pulmonary hypertension
8. Pulmonary embolism within past 3 months
9. Currently receiving extracorporeal life support (ECLS)
10. Chronic kidney disease receiving dialysis
11. Severe chronic liver disease with Child-Pugh score > 12
12. DNAR (Do Not Attempt Resuscitation) order in place
13. Treatment withdrawal imminent within 24 hours
14. Prisoners
15. Non-English speaking patients or those who do not adequately understand verbal or written information unless an interpreter is available.
16. Enrolled in a concomitant clinical trial of a medicinal product (other than antibiotics or anti-virals)
17. IgA deficiency

13 Recruitment

The population of interest in this study is severely ill COVID-19 patients with ARDS requiring ICU admission. Participants will be screened daily in the ICU by the Investigator or Clinical Research Nurse. The investigator will determine those who are eligible to participate in accordance with the inclusion/exclusion criteria.

14 Study procedures and schedule of assessments

14.1 Informed consent procedure

It is the responsibility of the Investigator, or a person delegated by the Investigator to obtain written informed consent from each subject. Given that participants are critically unwell, they will be unable to provide consent to participate prior to enrolment. The Investigator or nominee will obtain assent from the next of kin as soon as possible. In the event that a participant regains capacity, the Investigator or nominee with obtain explicit informed

consent to continue in the study. The informed consent/assent procedure will involve adequate explanation of the aims, methods, anticipated benefits and potential hazards of the study.

In the event that participants regain capacity they will be provided with a Regained Capacity Information Leaflet and will also need to sign the Regained Capacity Consent Form subject to a consent declaration by the Health Research Consent Declaration Committee.
“Adequate time” must be given for consideration by the patient and/or next of kin. The PI must record when the patient information leaflet (PIL) was given to the patient/next of kin.
The Investigator or designee will explain that the patients are under no obligation to enter the trial and that they can withdraw at any time during the trial, without having to give a reason for withdrawal.

Where any participant is enrolled and does not regain competency (due to their death or neurological impairment) the default position, subject to local laws and ethical review processes, will be that the enrolled person will continue to be a participant in the trial.
A copy of the signed Informed Consent form will be given to the participant/next of kin. **The original** signed form will be retained at the study site and a copy placed in the medical notes.
If new safety information results in significant changes in the risk/benefit assessment, the patient information leaflet will be reviewed and updated if necessary and subjects will be provided with the new PIL in a timely manner and consent obtained.

14.2 Randomisation procedures

Patients will be deemed suitable for inclusion by a member of the investigating team in the study at the bedside . Eligible patients will be randomised by unblinded trial personnel who are the only unblinded members of team involved in the study. Clinical details are relayed to the randomiser), who uses a software program to assign the patient to one of the 3 groups. The unblinded trial personnel hold the randomization list and treatment allocations. The Randomiser also makes up the IMP and will hand it to a blinded member of the trial team who will transfer it to the ICU or the ward to be administered by a blinded member of the trial team. The randomisation and IMP preparation process is detailed in a study specific SOP.

14.3 Unblinding

14.3.1 Emergency Unblinding

The study code should only be broken for valid medical or safety reasons e.g. in the case of a severe adverse event where it is necessary for the investigator or treating health care professional to know which treatment the patient is receiving before the participant can be treated. Subject always to clinical need, where possible, members of the research team should remain blinded.

The code breaks for the trial are held by the unblinded trial personnel who are the only unblinded members of the team involved in the study. These will be kept in a password protected file accessible only by the unblinded personnel.

In the event a code is required to be unblinded a formal request for unblinding will be made by the Investigator/treating health care professional.

If the person requiring the unblinding is a member of the Investigating team then a request to the holder of the code break envelope/list, or their delegate will be made and the unblinded information obtained.

If the person requiring the unblinding is not the CI/PI then that health care professional will notify the Investigating team that an unblinding is required for a trial subject and an assessment to unblind should be made in consultation with the clinical and research teams.

On receipt of the treatment allocation details the CI/PI or treating health care professional will deal with the participant's medical emergency as appropriate.

The CI/PI documents the breaking of the code and the reasons for doing so on the data collection tool, in the site file and medical records. It should also be documented at the end of the study in any final study report and/or statistical report.

The CI/Investigating team will notify the Sponsor in writing as soon as possible following the unblinding detailing the reasons. If the unblinding is the result of a serious adverse event SAE; the CI/PI must report the SAE to the sponsor within the timelines required by ICH GCP and the applicable legislations.

In view of the small sample size and relatively short duration of the study, a DSMB is not appropriate. However, after the 10th patient has been enrolled in the trial, an interim analysis will be carried out by the RCSI Statistician and data will be presented to two independent physicians, following which a decision regarding whether or not to proceed will be undertaken based on (1) safety and tolerability, and (2) the status of the primary endpoint.

14.3.2 Unblinding for the submission of SUSAR reports:

If a reported SAE appears to be a SUSAR, then the blinding should be broken (as per Section 14.3.1 above). Then three possibilities resulting from the procedure of unblinding must be considered:

- If the product administered to the subject is the tested investigational medicinal product, the case should be reported by the Sponsor as a SUSAR to the relevant Competent Authority and the relevant Ethics Committees.
- Events associated with placebo will usually not satisfy the criteria for a serious adverse drug reaction and therefore expedited reporting will not be required. However, if after unblinding, SUSARs are associated with placebo (e.g. reaction due to an excipient or impurity), it is the sponsor's responsibility to report such cases.

14.4 Screening Period

The following screening assessments will be undertaken to ensure that a patient meets the criteria for enrolment.
Planned screening assessments as part of clinical trial (all to occur within previous 2 days):

- Urine pregnancy test (for females of child bearing age)

The following assessments will occur as part of routine care, and all will be performed within the previous 5 days (*most recent results to be documented*):

- Physical examination
- Vital Signs (blood pressure, mean arterial pressure, heart rate, temperature, SaO_2, urine output, respiratory rate (where appropriate)
- Medical history (where possible)
- Medical chart review
- X-Ray
- Electrocardiogram
- Echocardiogram (where possible)
- Laboratory assessments (blood count, basic liver/renal/bone profile biochemistry, standard markers of inflammation such as C-reactive protein)
- Assessment of comorbidities
- Confirmation of diagnosis of ARDS, requiring (1) acute onset; (2) $PaO_2/FiO_2<200$ regardless of positive end-expiratory pressure; (3) bilateral infiltrates seen on frontal chest radiograph; (4) pulmonary artery wedge pressure of 18 mmHg or less when measured or no clinical evidence of left atrial hypertension
- Assessment of vasopressor requirement (target range 0.05-0.1µg/kg/min).

Of note:

- The baseline visit will occur no later than 36 hours after the screening visit
- The screening visit and baseline visit can occur on the same day
- During the treatment phase, visits will occur +/- 1 day of the scheduled date
- The follow up period will be for 1 week after the final dose of IMP
- The follow up visit after Early Discontinuation will be performed as soon as is possible.
- Patients will continue to be followed by the clinical team and the post-COVID-19 clinic thereafter.
- Due to the nature of the study population, screen failures will not be re-considered for enrolment.

14.5 Baseline assessments

- Eligibility determination following interpretation of results emanating from the screening process
- Repeat review of medical history (where possible), medical chart
- Repeat physical exam
- Review of assessments carried out since screening visit by clinical team as part of routine care (these include, but are not limited to, radiology, electrocardiogram, echocardiogram, complete blood count, basic liver/renal/bone profile biochemistry, standard markers of inflammation such as C-reactive protein)
- Vital Signs (blood pressure, mean arterial pressure, heart rate, temperature, SaO2, urine output, respiratory rate (where appropriate)
- Current ventilation settings
- Current vasopressor requirements
- Review of concomitant medications (within the previous 7 days)
- PaO2/FiO2 ratio
- Respiratory compliance
- SOFA score
- Circulating AAT level
- Isoelectric focusing on plasma samples to determine the phenotype.
- Randomization

NOTE: A plasma sample will be obtained for cytokine measurements following randomization (same day), and will be stored immediately

14.6 Treatment procedures

Active IMP (Prolastin)

Route of administration: intravenous infusion

Dose: 120mg/kg body weight intravenously once per week.

Infusion rate: 0.08 mL/kg/min as determined by patient response and comfort

Solution used for reconstitution: sterile water for injection

Post-infusion observation period: 30 minutes

Placebo (0.9% sodium chloride solution for infusion)

Route of administration: intravenous infusion

Infusion rate: 0.08 mL/kg/min as determined by patient response and comfort

Post-infusion observation period: 30 minutes

14.7 Subsequent assessments

Day 2 visit, day 7 visit, day 14, day 21 visit, day 28 visit

- Physical examination
- Medical chart review
- PaO2/FiO2
- Assessment of vasopressor requirement
- Respiratory compliance
- SOFA score
- Circulating AAT level
- Review of assessments carried out since previous study visit by clinical team as part of routine care (these include, but are not limited to, radiology, electrocardiogram, echocardiogram, complete blood count, basic liver/renal/bone profile biochemistry, standard markers of inflammation such as C-reactive protein)
- Vital Signs (blood pressure, mean arterial pressure, heart rate, temperature, SaO2, urine output, respiratory rate (where appropriate)
- Development of a clinical outcome measure (mortality, time on ventilator, shock, arrhythmia, AKI, need for RRT)

NOTE: plasma sample taken at baseline visit, and again at days 2, 7, 14, 21, 28, with cytokine levels assessed following the collection of all samples at the end of the trial.

14.8 Flowchart of study assessments

	Baseline [1]	Treatment Phase [2]					Follow Up
**Visit #**	**1**	**2**	**3**	**4**	**5**	**6**	**7**
Visit Type	Screening period	Baseline	Day 2 visit	Day 7 visit	Day 14 visit	Day 21 visit	Day 28 visit
Visit schedule	Day -5 to -1	Day 0	Day 2 (+/1 day)	Day 7 (+/1 day)	Day 14 (+/1 day)	Day 21 (+/1 day)	Day 28 (+/1 day)
Informed Consent (family member assent/regained capacity consent)	X	X	X[1]	X[1]	X[1]	X[1]	X[1]
Eligibility determination medical history review/ all systems physical exam/ pregnancy tests, laboratory assessments (blood & urine)/ECG/ECHO[2]/x-ray/ height/weight, age, sex, BMI	X	X[2]					
Physical exam	X	X	X	X	X	X	X
Medical Chart Review	X	X	X	X	X	X	X
Vital Signs Include BP, Pulse, Temp, Sa02, respiratory rate, urinary output, PaO2/FiO2, mean arterial pressure	X	X	X	X	X	X	X
Concomitant Medication review	X	X	X	X	X	X	X
PaO2/FiO2 ratio, respiratory compliance, SOFA score, circulating AAT level, plasma sample for storage (to be run in bulk at conclusion of each week).	X	X	X	X	X	X	X
Routine care blood results reviewed	X	X	X	X	X	X	X
Clinical outcomes recorded (mortality, time on ventilator in days, shock, AKI, need for RRT)	X	X	X	X	X	X	X
Randomisation		X					
IMP administration		X		X	X	X	
Adverse Events review	X	X	X	X	X	X	X

[1] Regained capacity consent must be attempted with the subject at each visit until it is obtained (*unless the subject does not regain capacity)*

[2] ECHO to be performed where possible

14.9 Methods

14.9.1 Laboratory procedures

The following samples will be processed at each hospital site laboratory:

- Investigations ordered by the clinical team as part of clinical need, including but not limited to complete blood count, basic liver/renal/bone profile biochemistry, standard markers of inflammation such as C-reactive protein

The following samples will be processed at each site and stored (*as per the SOP for processing, storing and shipping laboratory samples*). At the end of the trial, these samples will be shipped from each site and analysed at the RCSI respiratory research laboratory, a centralized laboratory for plasma processing from whole blood for the purpose of this study, located on the Beaumont Hospital campus:

- AAT level
- Plasma isolation
- Isoelectric focusing of plasma
- Cytokine measurements by ELISA

14.10 Definition of end of trial

The end of the trial will be defined as the last patient visit for the last patient enrolled to the trial.

14.11 Discontinuation/withdrawal of participants and 'stopping rules'

Participants will be able to withdraw from the study at any point without giving a reason. This will be explained in all information leaflets to the patient and/or next of kin.

15 Name and description of all drugs used in the trial

15.1 Treatment of subjects

Investigational product/treatment

Active IMP

Alpha1-proteinase inhibitor (human), commercially available as Prolastin, is a sterile, stable, lyophilized preparation of purified human alpha-1 antitrypsin (AAT). Prolastin is prepared from pooled human plasma from healthy donors by modification and refinements of the Cohn cold ethanol plasma fractionation technique followed by a purification process. Prolastin contains small amounts of other plasma proteins, which may include IgA, haptoglobin, alpha1-acid glycoprotein, lipoprotein A-1, and albumin. Reconstituted Prolastin contains no preservatives,

has a pH of 6.6 to 7.4, and is to be administered by the IV route. Each vial of lyophilized Prolastin will be reconstituted with 40 mL of Sterile Water for Injection (WFI).

Placebo IMP

The placebo to be used in the study is 0.9% sodium chloride solution for infusion ("normal saline").

Composition: 9.0 g/l sodium chloride (NaCl) in sterile water for injection. Each ml contains 9 mg sodium chloride. mmol/l: Na+ : 154 Cl- : 154. pH: 4.5-7.

Pharmaceutical form: Solution for infusion. Clear solution, free from visible particles.

Placebo and Active IMP

Preparations of placebo or active will be labelled, and then transferred to the ICU in a blinded fashion and will be administered at the bedside by a member of the clinical team.

15.2 Concomitant medication

There are no restrictions on medicine or treatment in this study. Prolastin has no known drug-drug interactions. The same applies to 0.9% sodium chloride solution for infusion.
Patients will receive usual care, which is to be founded on evidence-based medicine.

16 Investigational Medicinal Product

16.1 Name and description of investigational medicinal product(s)

Prolastin:

Alpha1-proteinase inhibitor (human), commercially available as Prolastin, is a sterile, stable, lyophilized preparation of purified human AAT. Prolastin is prepared from pooled human plasma from healthy donors by modification and refinements of the Cohn cold ethanol plasma fractionation technique followed by a purification process. Prolastin contains small amounts of other plasma proteins, which may include IgA, haptoglobin, alpha1-acid glycoprotein, lipoprotein A-1, and albumin. Reconstituted Prolastin contains no preservatives, has a pH of 6.6 to 7.4, and is to be administered by the IV route. Each vial of lyophilized Prolastin will be reconstituted with 40 mL of Sterile Water for Injection (WFI).

Placebo:

The placebo to be used in the study is 0.9% sodium chloride solution for infusion ("normal saline").

16.2 Name and description of each NIMP

Not applicable.

16.3 Summary of findings from non-clinical studies

In vitro and ex vivo studies:

The in vitro and ex vivo antiprotease and anti-inflammatory properties of plasma-purified AAT in general, and Prolastin specifically, are numerous. The reviewer is directed to the review by Bergin et al. [36].

Animal pharmacology studies [37].

Pharmacokinetics: The half-life of Prolastin administered intravenously in rabbits was determined to be 20.1 hours.

Pharmacodynamics: A series of studies was conducted in rats and rabbits to determine the effect of a single intravenous dose of Prolastin, 100 mg/kg, infused rapidly, 8 mL (168 mg)/min in rats and 6 mL (126 mg)/min in rabbits, on a number of clinical and biochemical parameters. Rats were studied both with and without an inhibitor of kininase II/angiotensin converting enzyme in order to potentiate any peptide-mediated cardiovascular effects which might be present. In rats, no significant cardiovascular or hematologic effects were observed, but a slight fall in fibrinogen 30 minutes following infusion of the Prolastin was noted. In rabbits, a marginal fall in leukocytes was observed, but this proved to be not statistically significant. No significant hematologic changes were detected.

Acute Toxicity: The acute toxicity of IV Prolastin was determined in mice, rats, and rabbits and compared to the acute toxicity of the excipient control substance. At an infusion rate of 3 mL/min, the LD50 of Prolastin in mice was 150±6 mL/kg (3,750 mg/kg) and that of the control was >156 mL/kg. In rabbits, there was no indication of any toxicity at the highest dose of Prolastin tested, 20.7 mL/kg, which was infused at a rate of 6 mL/kg (517 mg/kg) although one of three rabbits each in the groups receiving 6.9 mL and 20.7 mL/kg, respectively, of Prolastin died during the observation period. These two deaths were not related to administration of alpha1-PI. An additional three rabbits were administered alpha1-PI at a dose of 20.7 mL/kg without any sign of adverse effect throughout the 14-day observation period.

Subacute Toxicity: A series of rabbits also received Prolastin or excipient control substance, 9.1 mL/kg (227 mg/kg), administered intravenously at a rate of 6 mL/min, daily on five successive days. All rabbits in the study gained weight and there were no significant differences in weight gain on the 6th day or 33rd day of the study between animals receiving alpha1-PI compared to those receiving control substance. No significant hematologic abnormalities were noted on the 6th or 33rd days of the study following five consecutive days of administration of Prolastin. An unexplained decrease in the cholesterol level of animals receiving Prolastin was seen on day six in one series of animals but was not seen when repeated in another group. Two rabbits died during the course of the study, both of which were receiving Prolastin. One rabbit died on day 4, with diarrhea present, and its death was felt to be related to infection. The other rabbit died on day 27 (three weeks after

the infusion period) and histopathology revealed no probable cause of death. Overall, no effects directly ascribable to administration of Prolastin were detected in animals undergoing necropsy and histopathologic analysis on days 6 or 33 of the study.

16.4 Summary of findings from clinical studies

One of the primary functions of AAT in the human body is to irreversibly bind neutrophil elastase (NE) an omnivorous serine protease release by activated or disintegrating neutrophils that is responsible for destruction of lung tissue and drives airway inflammation [38-44]. NE levels and activity are markedly increased in the airways of patients with ARDS, and previous studies have shown that IV administration of Prolastin in humans results in augmented antielastase capacity of airway epithelial lining fluid, reduced NE activity in bronchoalveloar lavage fluid and decreased breakdown of lung tissue [1, 2, 4, 5]. While AAT chiefly inhibits NE, it also other exerts an antiprotease effect on chymotrypsin, cathepsin G (CathG) and proteinase 3 (PR3) [12]. The structure of the AAT is critical for its antiprotease activity and comprises 3 beta sheets (A, B and C), 9 alpha helices and a reactive center loop (RCL) at the C-terminal end [22].

In addition to its antiprotease effects [14-16], AAT is a potent anti-inflammatory and immunomodulator [17-21]. Of particular relevance to COVID-19, it has been shown to modulate suppress the production and activity of several key pro-inflammatory cytokines, including interleukin IL-1, IL-6, IL-8 and tumor necrosis factor (TNF)-α [17, 18, 22, 23], while preserving the production of the anti-inflammatory cytokine IL-10 [24]. Furthermore, we have also shown that abrupt cessation of plasma-purified AAT augmentation therapy for patients with AATD results in alterations to immune cell metabolism, inflammasome activation, dangerous increases in levels of these specific aforementioned pro-inflammatory cytokines, and subsequent progression to respiratory failure (results in press).

16.5 Summary of known and potential risks and benefits

<u>Benefits of Prolastin</u>:

- Clear anti-inflammatory effects in vitro, ex vivo and in vivo, both in the clinical trial setting and in clinical practice

- Clear anti-inflammatory effects in vitro, ex vivo and in vivo, both in the clinical trial setting and in clinical practice

- Shown to be protective against destruction of lung tissue

- Has been given safely to humans for over 40 years

- No known drug-drug interactions

Risks of Prolastin:

- Because Prolastin is made from human blood, it may carry the risk of transmitting infectious agents. The risk that such products will transmit an infectious agent has been reduced by screening plasma donors for prior exposure to certain viruses, by testing for the presence of certain current virus infections, and by inactivating and/or removing certain viruses.

- There is also the possibility that unknown infectious agents may be present in such products

- There will be an increase in plasma volume following intravenous administration of Prolastin. Caution should therefore be used in patients at risk for circulatory overload

- Adverse drug reactions are uncommon with Prolastin, both in the clinical trial and clinical practice setting

- In clinical studies, six reactions were observed with 517 infusions of Prolastin, or 1.16%. None of the reactions were severe. The adverse reactions reported included delayed fever (maximum temperature rise was 38.9°C, resolving spontaneously over 24 hours) occurring up to 12 hours following treatment (0.77%), lightheadedness (0.19%), and dizziness (0.19%). Mild transient leukocytosis and dilutional anemia several hours after infusion have also been noted.
- Additionally, since market entry of Prolastin, occasional reports of the following events have been received: flu-like symptoms, allergic-like reactions, dyspnea, tachycardia,shortness of breath, bronchospasm, wheezing, urticaria, back pain, clamminess, sweating, diarrhea, and fatigue. Less frequently, the following have also been reported: hypotension, anxiety, cyanosis, swelling of hands and feet, angio-, facial and lip oedema, nasal congestion, sinusitis, abdominal pains or cramps, pallor, and weakness. Rare cases of transient increase in blood pressure or hypertension and chest pain have also been reported.

16.6 Description and justification of route of administration and dosage

The route of administration and dosage described have been successfully used in both the clinical trial and clinical practice settings, and shown to be both safe and efficacious in high-risk populations. At present, AAT can only be delivered via the IV or aerosol route. The IV administration described avoids the exposure hazard related to aerosolized administration in COVID-19 patients, thereby reducing the risk to the clinical team.

16.7 Dosages, dosage modifications and method of administration

Group 1:

- Week 1: Prolastin 120mg/kg body weight IV over 60 minutes*

- Week 2: Placebo (same volume, same rate of infusion as for Prolastin)

- Week 3: Placebo (same volume, same rate of infusion as for Prolastin)

- Week 4: Placebo (same volume, same rate of infusion as for Prolastin)

Group 2:

- Week 1: Prolastin 120mg/kg body weight IV over 60 minutes*

- Week 2: Prolastin 120mg/kg body weight IV over 60 minutes*

- Week 3: Prolastin 120mg/kg body weight IV over 60 minutes*

- Week 4: Prolastin 120mg/kg body weight IV over 60 minutes*

Group 3:

- Week 1: Placebo (same volume, same rate of infusion as for Prolastin)

- Week 2: Placebo (same volume, same rate of infusion as for Prolastin)

- Week 3: Placebo (same volume, same rate of infusion as for Prolastin)

- Week 4: Placebo (same volume, same rate of infusion as for Prolastin)

The dose of Prolastin or placebo will not be modified during the study.

*The rate of infusion may be modified to accommodate clinical need (e.g. if the patient is clinically overloaded and cannot tolerate additional fluid). The infusion rate may be slowed down and given over a longer period of time if required but the IMP must be infused within 3 hours of preparation *(as per the Summary of Product Characteristics).*

Patients discharged from the ICU will receive the next scheduled dose on the ward that they are discharged to. Patients discharged from the hospital before receiving all scheduled doses will not receive the next scheduled dose.

16.8 Preparation and labelling of Investigational Medicinal Product

Preparation and labelling of the investigational medicinal products will be completed in accordance with the relevant guidelines and the Trial specific SOP for IMP preparation and administration. Administration of IMP to patients will be as per an IMP administration protocol and will be recorded in the IMP dispensing log and the medical notes. Each IMP infusion will be labelled with the approved clinical trial label. Text for labels used for IMP have been provided by the Sponsor (Appendix 1). The patency of IV access is formally tested pre-and-post administration of IMP.

16.9 Drug accountability

The precise amount of IMP required to conduct the study is already on-site and stored appropriately in the RCSI Smurfit building, Beaumont Hospital campus according to the storage conditions outlined in the SmPC. The IMP will be distributed to other clinical trial sites as per SOP 1: IMP Receipt, Preparation, Administration and Destruction.

16.10 Source of IMPs including placebo

Prolastin is supplied by Grifols pharmaceuticals free of charge, on compassionate grounds. 0.9% sodium chloride solution for infusion is supplied by the hospital pharmacy at each clinical trial site.

16.11 Dose modifications

In the event of clinical overload, the rate of infusion will be slowed, as guided by central venous pressure (CVP; all patients in the ICU will have CVP monitoring in situ as part of standard care).

In the event of an allergic, anaphylactoid or anaphylactic reaction to the IMP, the infusion will be discontinued and immediate review by immunology services will be arranged.

16.12 Assessment of compliance

N/A no compliance assessment for this study

16.13 Post-trial IMP arrangements

There are no arrangements in place for the IMP to be provided to trial subjects post trial participation. However, the manufacturer are willing to explore compassionate-use supply to these individuals should a clinical improvement occur in response to therapy.

17 Recording and reporting of adverse events and reactions

17.1 Definitions

Term	Definition
Adverse Event (AE)	Any untoward medical occurrence in a patient or clinical trial subject administered a medicinal product and which does not necessarily have a causal relationship with this treatment.
Adverse Reaction (AR)	Any untoward and unintended response in a subject to an investigational medicinal product which **is related** to any dose administered to that subject.<br>*This includes medication errors, uses outside of protocol (including misuse and abuse of product)*

Serious adverse event (SAE), serious adverse reaction (SAR) or unexpected serious adverse reaction	Any adverse event that:<br>• results in death,<br>• is life-threatening,<br>• requires hospitalisation or prolongation of existing hospitalisation,<br>• results in persistent or significant disability or incapacity, or<br>• consists of a congenital anomaly or birth defect
Important Medical Event	These events may jeopardise the subject or may require an intervention to prevent one of the above characteristics/consequences. Such events should also be considered 'serious'.
SUSAR	Suspected Unexpected Serious Adverse Reaction<br>A serious adverse reaction the nature and severity of which is not consistent with the information about the medicinal product in question set out:<br>(a) in the case of a product with a marketing authorization, in the summary of product characteristics for that product,<br>(b) in the case of any other investigational medicinal product, in the investigator's brochure relating to the trial in question.

17.2 Recording adverse events

As this trial is recruiting in a population that is already in a life-threatening situation, it is expected that many of the patients will experience multiple AEs. In general, events that are expected in this population (i.e. events in keeping with the underlying condition) should not be reported as AEs / SAEs as they represent disease progression. All clinical events will be recorded in the medical record as per normal practice.

The following adverse events will be collected:

A. Adverse events of special interest (AESI) occurring within 2 hours of the start of infusion:

1. New ventricular tachycardia (persistent tachycardia lasting > 30 minutes)*
2. Clinical scenario consistent with infusion reaction (e.g. urticaria, angioedema, new bronchospasm, anaphylaxis).
3. Persistent hypotension lasting > 30 minutes and requiring intervention with vasopressors*
4. Persistent hypertension lasting > 30 minutes and requiring intervention with hypotensive agents*
5. Any event occurring within this time period which is considered by the investigator to be atypical in a patient with ARDS.

**multiple episodes will be captured as one Adverse Event (AE)*

B. After the initial infusion period and until the patient is discharged from hospital, the following events will be recorded:

AEs that are (in the opinion of the Investigator):
- Atypical for a patient with ARDS
- Unexpectedly severe or frequent for a patient with ARDS.
- In line with the side effects outlined in the SmPC for Prolastin and not otherwise explained by the treated condition.

Note – typical complications of ARDS, ventilation or treatment in the intensive care unit (including but not limited to multi-organ failure, pneumonia, urinary tract infection, sepsis etc.) will not be considered AEs.

Events which meet the criteria in A or B will be recorded as AEs. AEs that also meet criteria for seriousness will be recorded as SAEs and will be reported to the Sponsor as outlined in Section 17.4.

The investigator should attempt, if possible, to establish a diagnosis based on the subject's signs and symptoms. Where there is no diagnosis, symptoms will be recorded. When a diagnosis for the reported signs or symptoms is known, the investigator should report the diagnosis as the AE, rather than reporting the individual symptoms.

All adverse events (i.e. AEs) will be recorded in the medical records, an AE or SAE form (as appropriate) and/or in the trial database (spreadsheet) following enrolment, in accordance with the protocol. All AEs should be treated appropriately. The following information will be recorded: description, date of onset and end date, severity, assessment of relatedness to the study medication, and action taken and outcome. Follow-up information should be provided as necessary.

If the investigator suspects that the subjects' disease has progressed faster due to the administration of the IMP, then he will record and report this as an AE. It will be left to the investigator's clinical judgment whether or not an AE is of sufficient severity to require the subject's removal from treatment. A subject may also voluntarily withdraw from treatment due to what he or she perceives as an intolerable AE. If either of these occurs, the subject must undergo an end-of-study assessment and be given appropriate care under medical supervision until symptoms cease or the condition becomes stable.

17.3 Assessments of Adverse Events

Each adverse event will be assessed for the following criteria:

17.3.1 Severity

Category	Definition
Mild	The adverse event does not interfere with the volunteer's daily routine, and does not require intervention; it causes slight discomfort
Moderate	The adverse event interferes with some aspects of the volunteer's routine, or requires intervention, but is not damaging to health; it causes moderate discomfort
Severe	The adverse event results in alteration, discomfort or disability which is clearly damaging to health

17.3.2 Causality

The assessment of relationship of adverse events to the administration of IMP is a clinical decision based on all available information at the time of the completion of the case report form. The following categories will be used to define the causality of the adverse event:

Category	Definition
Definitely:	There is clear evidence to suggest a causal relationship, and other possible contributing factors can be ruled out.
Probably:	There is evidence to suggest a causal relationship, and the influence of other factors is unlikely
Possibly	There is some evidence to suggest a causal relationship (e.g. the event occurred within a reasonable time after administration of the trial medication). However, the influence of other factors may have contributed to the event (e.g. the patient's clinical condition, other concomitant events).
Unlikely	There is little evidence to suggest there is a causal relationship (e.g. the event did not occur within a reasonable time after administration of the trial medication). There is another reasonable explanation for the event (e.g. the patient's clinical condition, other concomitant treatments).
Not related	There is no evidence of any causal relationship.

17.3.3 Expectedness

Category	Definition

Expected	An adverse event that is classed in nature as serious and which is consistent with the information about the IMP listed in the Investigator Brochure (or SmPC if Licensed IMP)
Unexpected	An adverse event that is classed in nature as serious and which is not consistent with the information about the IMP listed in the Investigator Brochure (or SmPC if Licensed IMP)

The reference document to be used by the Sponsor to assess expectedness of the event against the IMP. The reference document is the Summary of Product Characteristics (SmPC) for Prolastin (Appendix 2). It is important to note that SAEs that are not related to the IMP do not require an expectedness assessment and will not be reported to the Sponsor.

17.3.4 Seriousness

The Principal Investigator (PI) should make an assessment of seriousness as defined in section 17.1.

Collection, recording and reporting of reportable serious adverse events to the sponsor will be completed according to the RCSI Expedited Reporting SOP (Appendix 3)

17.4 Procedures for recording and reporting Serious Adverse Events

All serious adverse events will be recorded by the site staff in the hospital notes, the database and on the sponsor's SAE form.

The Principal Investigator (PI) or appropriate designee is responsible for reporting all SAEs to RCSI Pharmacovigilance (Pharmacovigilance@rcsi.ie) within 24 hours of first becoming aware of the event (as per the RCSI Sponsor SOP on Expedited Safety Reporting).

SAEs will be collected from the time of the subject's enrolment until the final study visit. SAEs that continue beyond the normal collection period (i.e., are ongoing at the time a subject exits the study) will be followed until resolution or until stabilized with sequelae. SAEs that begin after the subject's participation in the study is complete, but that the PI considers to be related to study drug, may be reported at any time.

Reporting to the sponsor will be completed as per the sponsor's pharmacovigilance SOP (RCSI SOP for Expedited Safety Reporting).

17.4.1 Notification of deaths

Death will not be captured as a Serious Adverse Event. Death will be captured as an outcome. However, the cause of death may be captured as a Serious Adverse Event, unless it is judged to be related to disease progression as detailed above.

17.4.2 Reporting SUSARs

The sponsor will notify the main REC and competent authority of all SUSARs. SUSARs that are fatal or life-threatening must be notified to the CA and REC within 7 calendar days after the sponsor has learned of them. Other SUSARs must be reported to the REC and CA within 15 calendar days after the sponsor has learned of them.

17.4.3 Development Safety Update Reports

The sponsor will provide the main REC and the competent authority with Development Safety Update Reports (DSUR) which will be written in conjunction with the trial team and the Sponsorship office. The report will be submitted within 60 days of the Developmental International Birth Date (DIBD) of the trial each year until the trial is declared ended.

17.4.4 Annual progress reports

An annual progress report (APR) will be submitted to the REC within 30 days of the anniversary date on which the favourable opinion was given, and annually until the trial is declared ended. The sponsor will prepare the APR.

17.4.5 Pregnancy

The effects of Prolastin therapy during pregnancy have not been studied in a clinical trial. However, Prolastin has been received by pregnant women since becoming commercial available. To date, no adverse events have been published in the literature.
0.9% sodium chloride solution for infusion is given routinely to pregnant patients in the ICU setting. Participants will undergo a formal pregnancy test at the screening visit.
Pregnant and breastfeeding women are excluded from the study. If a pregnancy does occur during the trial, it will be reported to the Sponsor according to the RCSI Expedited Reporting SOP (Appendix 3).

17.4.6 Overdose

To date, there have been no reported cases of overdose for Prolastin or other plasma-purified AAT product. No data are available in regard to overdosage in humans.
While not constituting and overdose, clinical overload from excessive volume should be avoided. Avoidance of overload will be facilitated by monitoring of clinical signs such as venous pressure and by slowing the rate of infusion as necessary. Overload may also be alleviated using diuretic therapy, a standard component of the management of patients with ARDS.

17.4.7 Reporting Urgent Safety Measures

If any urgent safety measures are taken the PI/Sponsor shall immediately and in any event no later than 3 days from the date the measures are taken, give written notice to the competent authority and the relevant REC of the measures taken and the circumstances giving rise to those measures.

17.5 The type and duration of the follow-up of subjects after adverse events.

Any SAR related to the IMP will be reported to the Sponsor irrespective of how long after IMP administration the reaction has occurred. AEs considered related to the study medication as judged by an investigator or the sponsor will be followed until resolution or until the event is considered stable. All related AEs that result in a subject's withdrawal from the study or are present at the end of the study, should be followed up until a satisfactory resolution occurs

17.5.1 Notification of Serious Breaches to GCP and/or the protocol

A "serious breach" is a breach which is likely to effect to a significant degree –

(a) the safety or physical or mental integrity of the subjects of the trial; or

(b) the scientific value of the trial.

The sponsor of a clinical trial shall notify the competent authority in writing of any serious breach of –

(a) the conditions and principles of GCP in connection with that trial; or (b) the protocol relating to that trial, as amended from time to time, within 7 days of becoming aware of that breach.

The sponsor will be notified immediately of any case where the above definition applies during the trial conduct phase. The sponsor's SOP on the 'Notification of violations, urgent safety measures and serious breaches' will be followed.

18 Data management and quality assurance

18.1 Confidentiality

All data will be handled in accordance with the applicable Data Protection legislation

Trial documentation will not bear the subject's name or other personal identifiable data. A participant study number will be used for identification.

18.2 Data collection tools and source document identification

Data will be pseudonymised. The pseudonymised data will be stored on an encrypted and password protected electronic file (spreadsheet). The password will be held by members of the trial team only so that stored data can only be accessed by those on the trial delegation log. The investigator site file will be maintained by the principal investigator in a secure, access controlled cabinet. Source documentation will include paper and electronic heath records.

It will be the responsibility of the investigator to ensure the accuracy of all data entered in the trial database (spreadsheet). The delegation log will identify all those

personnel with responsibilities for data collection and handling, including those who have access to the trial database.

18.3 Data handling and analysis

A spreadsheet will be used for data entry.

The medical chart will act as source data. The trial data will be pseudonymised and inputted onto a spreadsheet that is encrypted and password protected. This file will be stored on a secure server within RCSI (see Section 18.2). None of the pseudonymised data will be shared with third parties.

The study investigators will be responsible for data entry and quality. The study statistician will perform data analysis (see Section 20.3)

19 Record keeping and archiving

Archiving will be authorised by the Sponsor following submission of the end of study report.

Records will be retained at a secure archiving facility following end of study report.

Trial data will be retained for:

· Essential documents : 10 years after completion of trial

· the trial database: 10 years after completion of trial

The chief investigator is responsible for the secure archiving of Investigator Site File essential trial documents and the trial database.

Destruction of essential documents will require authorisation from the Sponsor.

20 Statistical Considerations

Dr. Fiona Boland is the trial statistician who will be responsible for all statistical aspects of the trial from design through to analysis and dissemination

20.1 Outcomes

20.1.1 Primary outcomes

The primary effectiveness outcome measure, a continuous variable, is the change in IL-6 in plasma at 7 days as measured by ELISA.

20.1.2 Secondary outcomes

Safety and tolerability of IMP in the respective groups, as defined by the number of SEAs and AEs, binary variable

- PaO2/FiO2 ratio, continuous variable

Respiratory compliance, continuous variable

- Sequential organ failure assessment (SOFA) score, continuous variable
- Mortality, binary variable
- Time on ventilator in days, continuous variable
- Circulating AAT levels as measured by nephelometry, continuous variable
- Plasma levels of IL-1β as measured by ELISA, continuous variable
- Plasma levels of IL-8 as measured by ELISA, continuous variable
- Plasma levels of IL-10 as measured by ELISA, continuous variable
- Plasma levels levels of soluble TNF receptor 1 (sTNFR1, a surrogate marker for TNF-α) as measured by ELISA, continuous variable
- Development of shock, defined for the purpose of this study as life-threatening organ dysfunction caused by a dysregulated response to infection, with critical reduction in tissue perfusion and acute failure of multiple organs, including the lungs, kidneys, and liver, binary variable
- Acute kidney injury defined as an abrupt sustained rise in urea and creatinine, binary variable
- Need for renal replacement therapy, binary variable
- Clinical relapse, as defined by the need for readmission to the ICU or a marked decline in PaO2/FiO2 or development of shock or mortality following a period of sustained clinical improvement, binary variable
- Secondary bacterial pneumonia as defined by the combination of radiographic findings and sputum/airway secretion microscopy and culture, binary variable

20.2 Sample size and recruitment

20.2.1 Sample size calculation

As this is a pilot study a formal sample size calculation has not been conducted. Sample sizes of between 12 per group and 25 per group have been recommended variously for pilot studies [45-47]. Following these broad recommendations and clinical experience, we chose a recruitment sample size of 36 (12 per group) to assess the safety and tolerability, and to explore the biological effect. The study sample size is sufficient to demonstrate a significant difference in patients receiving Prolastin versus patients receiving placebo.

20.2.2 Planned recruitment rate

It is estimated that all participants will be recruited within 12 months.

20.3 Statistical analysis plan

20.3.1 Summary of baseline data and flow of patients

The data will be summarized using appropriate descriptive statistics with respect to demographic and baseline characteristics. A consort flow diagram will be presented also.

20.3.2 Primary outcome analysis

Efficacy observations and measurements and safety observations and measurements will be summarized using appropriate descriptive statistics (n, mean, standard deviation, median, minimum, maximum) for quantitative data and contingency tables (frequencies and percentages) for qualitative data.
Participants will be analysed according to the planned treatment they have been assigned to. No imputation of missing data is planned. All withdrawals, missing and spurious data will be explored and reported in the final report.

20.3.3 Secondary outcome analysis

As outlined above for the primary outcome measures, a similar approach will be conducted here. Appropriate descriptive statistics will be used to explore the data. Estimates and 95% confidence intervals will be reported.

20.3.4 Sensitivity and other planned analyses

No other analyses planned.

20.4 Randomisation methods

Randomisation will take place following baseline data collection. The randomisation list for each site will be prepared by the study statistician and will be communicated to the unblinded trial personnel responsible for randomisation. Participants will be randomised to study arms stratified by age only. Block randomisation is applied at site level aimed at achieving equal numbers in each study arm as far as possible.

20.5 Interim analysis

An interim analysis of cytokine levels taken at 7 days may be conducted at after 10 patients have been enrolled.

A 24-hour contact number will be available in the circumstances when unblinding is required. The principal investigator will document the breaking of the code, and the reasons for doing so. It is at the discretion of the treating clinician whether unblinding should occur. Overall, it is felt that unblinding will be an extremely unlikely event. Any compromises in blinding will be reported in the trial conclusion. Otherwise, study unblinding will only take place once the statistical analysis plan has been agreed by the trial team and the final database has been locked.

20.6 Other statistical considerations

As outlined above, a full SAP will be prepared prior to final analysis. Any deviations from this SAP will be described and justified in the final report.
Fiona Boland is the trial statistician who will be responsible for all statistical aspects of the trial from design through to analysis and dissemination.
Due to the urgent nature and rapid progression of the crisis, the study may start recruitment before the SAP is finalised. The SAP will be written, reviewed and signed off as soon as is practical during the study.

21 Name of Committees involved in trial

Ethics Committee Beaumont Hospital Beaumont Road Dublin 9.

Health Research Consent Declaration Committee.

22 Direct Access to Source Data/Documents

The investigator(s)/ institution(s) will permit trial-related monitoring, audits, REC review, and regulatory inspection(s), providing direct access to source data/documents. Trial participants are informed of this during the informed consent discussion. Participants will consent to provide access to their medical notes.

23 Ethics and regulatory requirements

The sponsor will ensure that the trial protocol, patient information leaflet, informed consent form, GP letter and submitted supporting documents have been approved by the appropriate competent authority and a research ethics committee, prior to any patient recruitment. The protocol and all agreed substantial protocol amendments, will be documented and submitted for ethical and regulatory approval prior to implementation.

Before the site can enrol patients into the trial, the Principal Investigator or designee must apply to the hospital for permission to conduct the study and be granted written permission. It is the responsibility of the Chief Investigator/ Principal Investigator or designee at each site to ensure that all subsequent amendments gain the necessary approval. This does not affect the individual clinician’s responsibility to take immediate action if thought necessary to protect the health and interest of individual patients (see section 17.4.7 for reporting urgent safety measures).

Within 90 days after the end of the trial, the CI/Sponsor will ensure that the main REC and the competent authority is notified that the trial has finished. If the trial is terminated prematurely, those reports will be made within 15 days after the end of the trial.

24 Monitoring requirement for the trial

The sponsor will assign an independent monitor who will visit the investigator sites intermittently to validate compliance of the protocol to the GCP, the maintenance of the study related records, and the extensiveness and accuracy of a proportion of trial spreadsheet entries compared to source data (medical chart). The investigator will co-operate with the monitor to ensure that any potential discrepancies are resolved.

Monitoring procedures include a remote site initiation visit designed to clarify all prerequisites before the trial commences at the site, interim site monitoring visits and study close-out visits. The study will be monitored by scheduled visits to site and on-going communication via telephone, video calls and e-mail with the investigator and research nurse.

During on-site and remote monitoring visits the monitor may review; original patient records for the patient group; trial spreadsheet entries, drug accountability records; investigator site file and document retention. Study procedures will be observed by the monitor and any issues will be discussed with the PI or designee as necessary.

Direct access to all source documents, where possible, must be guaranteed by the PI, who must provide support at all times for these activities.

25 Finance

An application has been submitted to the HRB to support the trial.

26 Insurance

"RCSI holds insurance against claims from participants for injury caused by their participation in the clinical trial. However, as this clinical trial is being carried out in hospitals, each hospital continues to have a duty of care to the participant of the clinical trial. RCSI does not accept liability for any breach in the hospital's duty of care, or any negligence on the part of hospital employees. This applies whether the hospital is an HSE hospital or otherwise.
Beaumont Hospital, Galway University Hospital, St James's University Hospital and Mater Misericordiae University Hospital selected to participate in this clinical trial shall provide clinical indemnity cover or relevant insurance for harm caused by their employees.

27 Publication policy

All proposed publications will be discussed with Sponsor prior to publishing other than those presented at scientific forums/meetings. Please refer to the RCSI publication policy.

28 Statement of compliance

The trial will be conducted in compliance with the approved protocol, the ROI Regulations, ICH GCP E6 (R2) and the applicable regulatory requirement(s).

29 Appendices

Appendix 1: Sample IMP Label
Appendix 2: SmPC Prolastin
Appendix 3: RCSI SOP for expedited safety reporting

30 References

1. Campos MA, Geraghty P, Holt G, et al. The Biological Effects of Double-Dose Alpha-1 Antitrypsin Augmentation Therapy. A Pilot Clinical Trial. *Am J Respir Crit Care Med*. 2019; 200: 318-26.
2. Chapman KR, Burdon JG, Piitulainen E, et al. Intravenous augmentation treatment and lung density in severe alpha1 antitrypsin deficiency (RAPID): a randomised, double-blind, placebo-controlled trial. *Lancet*. 2015; 386: 360-8.
3. Gadek JE, Klein HG, Holland PV and Crystal RG. Replacement therapy of alpha 1-antitrypsin deficiency. Reversal of protease-antiprotease imbalance within the alveolar structures of PiZ subjects. *J Clin Invest*. 1981; 68: 1158-65.
4. McElvaney NG, Burdon J, Holmes M, et al. Long-term efficacy and safety of alpha1 proteinase inhibitor treatment for emphysema caused by severe alpha1 antitrypsin deficiency: an open-label extension trial (RAPID-OLE). *Lancet Respir Med*. 2017; 5: 51-60.
5. Wewers MD, Casolaro MA, Sellers SE, et al. Replacement therapy for alpha 1-antitrypsin deficiency associated with emphysema. *N Engl J Med*. 1987; 316: 1055-62.
6. Huang C, Wang Y, Li X, et al. Clinical features of patients infected with 2019 novel coronavirus in Wuhan, China. *Lancet*. 2020; 395: 497-506.
7. Chen N, Zhou M, Dong X, et al. Epidemiological and clinical characteristics of 99 cases of 2019 novel coronavirus pneumonia in Wuhan, China: a descriptive study. *Lancet*. 2020; 395: 507-13.
8. Wu C, Chen X, Cai Y, et al. Risk Factors Associated With Acute Respiratory Distress Syndrome and Death in Patients With Coronavirus Disease 2019 Pneumonia in Wuhan, China. *JAMA Intern Med*. 2020.
9. Wu Z and McGoogan JM. Characteristics of and Important Lessons From the Coronavirus Disease 2019 (COVID-19) Outbreak in China: Summary of a Report of 72314 Cases From the Chinese Center for Disease Control and Prevention. *JAMA*. 2020.
10. Niederman MS, Richeldi L, Chotirmall SH and Bai C. Rising to the Challenge of the Novel SARS-coronavirus-2 (SARS-CoV-2): Advice for Pulmonary and Critical Care and an Agenda for Research. *Am J Respir Crit Care Med*. 2020.
11. Cao B, Wang Y, Wen D, et al. A Trial of Lopinavir-Ritonavir in Adults Hospitalized with Severe Covid-19. *N Engl J Med*. 2020.
12. Greene CM and McElvaney NG. Proteases and antiproteases in chronic neutrophilic lung disease - relevance to drug discovery. *Br J Pharmacol*. 2009; 158: 1048-58.
13. Elliott PR, Pei XY, Dafforn TR and Lomas DA. Topography of a 2.0 A structure of alpha1-antitrypsin reveals targets for rational drug design to prevent conformational disease. *Protein Sci*. 2000; 9: 1274-81.
14. Matheson NR, Wong PS, Schuyler M and Travis J. Interaction of human alpha-1-proteinase inhibitor with neutrophil myeloperoxidase. *Biochemistry*. 1981; 20: 331-6.
15. Ogushi F, Fells GA, Hubbard RC, Straus SD and Crystal RG. Z-type alpha 1-antitrypsin is less competent than M1-type alpha 1-antitrypsin as an inhibitor of neutrophil elastase. *J Clin Invest*. 1987; 80: 1366-74.
16. Sinden NJ, Baker MJ, Smith DJ, Kreft JU, Dafforn TR and Stockley RA. alpha-1-antitrypsin variants and the proteinase/antiproteinase imbalance in chronic obstructive pulmonary disease. *Am J Physiol Lung Cell Mol Physiol*. 2015; 308: L179-90.
17. Bergin DA, Reeves EP, Meleady P, et al. alpha-1 Antitrypsin regulates human neutrophil chemotaxis induced by soluble immune complexes and IL-8. *J Clin Invest*. 2010; 120: 4236-50.

18. Bergin DA, Reeves EP, Hurley K, et al. The circulating proteinase inhibitor alpha-1 antitrypsin regulates neutrophil degranulation and autoimmunity. *Sci Transl Med*. 2014; 6: 217ra1.
19. Hurley K, Lacey N, O'Dwyer CA, et al. Alpha-1 antitrypsin augmentation therapy corrects accelerated neutrophil apoptosis in deficient individuals. *J Immunol*. 2014; 193: 3978-91.
20. O'Dwyer CA, O'Brien ME, Wormald MR, et al. The BLT1 Inhibitory Function of alpha-1 Antitrypsin Augmentation Therapy Disrupts Leukotriene B4 Neutrophil Signaling. *J Immunol*. 2015; 195: 3628-41.
21. Jonigk D, Al-Omari M, Maegel L, et al. Anti-inflammatory and immunomodulatory properties of alpha1-antitrypsin without inhibition of elastase. *Proc Natl Acad Sci U S A*. 2013; 110: 15007-12.
22. Pott GB, Chan ED, Dinarello CA and Shapiro L. Alpha-1-antitrypsin is an endogenous inhibitor of proinflammatory cytokine production in whole blood. *J Leukoc Biol*. 2009; 85: 886-95.
23. McCarthy C, Dunlea DM, Saldova R, et al. Glycosylation Repurposes Alpha-1 Antitrypsin for Resolution of Community-acquired Pneumonia. *Am J Respir Crit Care Med*. 2018; 197: 1346-9.
24. Janciauskiene SM, Nita IM and Stevens T. Alpha1-antitrypsin, old dog, new tricks. Alpha1-antitrypsin exerts in vitro anti-inflammatory activity in human monocytes by elevating cAMP. *J Biol Chem*. 2007; 282: 8573-82.
25. Murphy MP, McEnery T, McQuillan K, et al. Alpha-1 antitrypsin therapy modulates the neutrophil membrane proteome and secretome. *Eur Respir J*. 2020.
26. O'Brien ME, Fee L, Browne N, et al. Activation of complement component 3 is associated with airways disease and pulmonary emphysema in alpha-1 antitrypsin deficiency. *Thorax*. 2020.
27. Zhou X, Shapiro L, Fellingham G, Willardson BM and Burton GF. HIV replication in CD4+ T lymphocytes in the presence and absence of follicular dendritic cells: inhibition of replication mediated by alpha-1-antitrypsin through altered IkappaBalpha ubiquitination. *J Immunol*. 2011; 186: 3148-55.
28. Munch J, Standker L, Adermann K, et al. Discovery and optimization of a natural HIV-1 entry inhibitor targeting the gp41 fusion peptide. *Cell*. 2007; 129: 263-75.
29. Greulich T, Chlumsky J, Wencker M, et al. Safety of biweekly alpha1-antitrypsin treatment in the RAPID programme. *Eur Respir J*. 2018; 52.
30. Hubbard RC, Sellers S, Czerski D, Stephens L and Crystal RG. Biochemical efficacy and safety of monthly augmentation therapy for alpha 1-antitrypsin deficiency. *JAMA*. 1988; 260: 1259-64.
31. Stoller JK, Fallat R, Schluchter MD, et al. Augmentation therapy with alpha1-antitrypsin: patterns of use and adverse events. *Chest*. 2003; 123: 1425-34.
32. Wencker M, Banik N, Buhl R, Seidel R and Konietzko N. Long-term treatment of alpha1-antitrypsin deficiency-related pulmonary emphysema with human alpha1-antitrypsin. Wissenschaftliche Arbeitsgemeinschaft zur Therapie von Lungenerkrankungen (WATL)-alpha1-AT-study group. *Eur Respir J*. 1998; 11: 428-33.
33. Blanco I, Lara B and de Serres F. Efficacy of alpha1-antitrypsin augmentation therapy in conditions other than pulmonary emphysema. *Orphanet J Rare Dis*. 2011; 6: 14.
34. Franciosiz AN, McCarthy C, Carroll TP and McElvaney NG. Unusual Acute Sequelae of alpha1-Antitrypsin Deficiency: A Myriad of Symptoms With One Common Cure. *Chest*. 2015; 148: e136-e8.
35. Greene C, Taggart C, Lowe G, Gallagher P, McElvaney N and O'Neill S. Local impairment of anti-neutrophil elastase capacity in community-acquired pneumonia. *J Infect Dis*. 2003; 188: 769-76.

36. Bergin DA, Hurley K, McElvaney NG and Reeves EP. Alpha-1 antitrypsin: a potent anti-inflammatory and potential novel therapeutic agent. *Arch Immunol Ther Exp (Warsz)*. 2012; 60: 81-97.
37. https://www.hpra.ie/img/uploaded/swedocuments/LicenseSPC_PA1405-002-001_11082017160036.pdf
38. Berger M, Sorensen RU, Tosi MF, Dearborn DG and Doring G. Complement receptor expression on neutrophils at an inflammatory site, the Pseudomonas-infected lung in cystic fibrosis. *J Clin Invest*. 1989; 84: 1302-13.
39. Birrer P, McElvaney NG, Rudeberg A, et al. Protease-antiprotease imbalance in the lungs of children with cystic fibrosis. *Am J Respir Crit Care Med*. 1994; 150: 207-13.
40. Cantin AM, Berthiaume Y, Cloutier D and Martel M. Prolastin aerosol therapy and sputum taurine in cystic fibrosis. *Clin Invest Med*. 2006; 29: 201-7.
41. Geraghty P, Rogan MP, Greene CM, et al. Neutrophil elastase up-regulates cathepsin B and matrix metalloprotease-2 expression. *J Immunol*. 2007; 178: 5871-8.
42. Hartl D, Latzin P, Hordijk P, et al. Cleavage of CXCR1 on neutrophils disables bacterial killing in cystic fibrosis lung disease. *Nat Med*. 2007; 13: 1423-30.
43. McElvaney OJ, Gunaratnam C, Reeves EP and McElvaney NG. A specialized method of sputum collection and processing for therapeutic interventions in cystic fibrosis. *J Cyst Fibros*. 2019; 18: 203-11.
44. Tosi MF, Zakem H and Berger M. Neutrophil elastase cleaves C3bi on opsonized pseudomonas as well as CR1 on neutrophils to create a functionally important opsonin receptor mismatch. *J Clin Invest*. 1990; 86: 300-8.
45. Lancaster GA, Dodd S and Williamson PR. Design and analysis of pilot studies: recommendations for good practice. *J Eval Clin Pract*. 2004; 10: 307-12.
46. Moore CG, Carter RE, Nietert PJ and Stewart PW. Recommendations for planning pilot studies in clinical and translational research. *Clin Transl Sci*. 2011; 4: 332-7.
47. Sim J and Lewis M. The size of a pilot study for a clinical trial should be calculated in relation to considerations of precision and efficiency. *J Clin Epidemiol*. 2012; 65: 301-8.

Covid 19 AAT Trial Protocol Version 3 23Nov2020

Final Audit Report 2020-12-09

Created:	2020-12-08
By:	Mandy Jackson (mandyjackson@rcsi.com)
Status:	Signed
Transaction ID:	CBJCHBCAABAAaxMlzonQZ9vt4mLsVAmF6rwikVqt9eT_

"Covid 19 AAT Trial Protocol Version 3 23Nov2020" History

Document created by Mandy Jackson (mandyjackson@rcsi.com)
2020-12-08 - 8:46:35 PM GMT- IP address: 37.228.215.123

Document emailed to Gerard Curley (gercurley@rcsi.ie) for signature
2020-12-08 - 8:47:12 PM GMT

Email viewed by Gerard Curley (gercurley@rcsi.ie)
2020-12-08 - 9:00:31 PM GMT- IP address: 78.17.194.14

Document e-signed by Gerard Curley (gercurley@rcsi.ie)
Signature Date: 2020-12-08 - 9:01:57 PM GMT - Time Source: server- IP address: 78.17.194.14

Document emailed to Maurice Dowling (mauricedowling@rcsi.ie) for signature
2020-12-08 - 9:01:59 PM GMT

Email viewed by Maurice Dowling (mauricedowling@rcsi.ie)
2020-12-09 - 7:57:46 AM GMT- IP address: 104.47.6.254

Document e-signed by Maurice Dowling (mauricedowling@rcsi.ie)
Signature Date: 2020-12-09 - 7:58:00 AM GMT - Time Source: server- IP address: 109.77.178.239

Agreement completed.
2020-12-09 - 7:58:00 AM GMT
